# Impaired Detoxification of Trans, Trans‐2,4‐Decadienal, an Oxidation Product from Omega‐6 Fatty Acids, Alters Insulin Signaling, Gluconeogenesis and Promotes Microvascular Disease

**DOI:** 10.1002/advs.202302325

**Published:** 2023-12-07

**Authors:** Xin Qian, Stephan Klatt, Katrin Bennewitz, David Philipp Wohlfart, Bowen Lou, Ye Meng, Michael Buettner, Gernot Poschet, Jakob Morgenstern, Thomas Fleming, Carsten Sticht, Ingrid Hausser, Ingrid Fleming, Julia Szendroedi, Peter Paul Nawroth, Jens Kroll

**Affiliations:** ^1^ Department of Vascular Biology European Center for Angioscience (ECAS) Medical Faculty Mannheim Heidelberg University 68167 Mannheim Germany; ^2^ Institute for Vascular Signaling Centre for Molecular Medicine Goethe‐University am Main 60590 Frankfurt Germany; ^3^ The German Centre for Cardiovascular Research (DZHK) Partner site RheinMain 60590 Frankfurt Germany; ^4^ Bone Marrow Transplantation Center The First Affiliated Hospital Zhejiang University School of Medicine Hangzhou 310003 China; ^5^ Metabolomics Core Technology Platform Centre for Organismal Studies Heidelberg University 69120 Heidelberg Germany; ^6^ Department of Internal Medicine I and Clinical Chemistry Heidelberg University Hospital 69120 Heidelberg Germany; ^7^ NGS Core Facility Medical Faculty Mannheim Heidelberg University 68167 Mannheim Germany; ^8^ Institute of Pathology IPH EM Lab Heidelberg University Hospital 69120 Heidelberg Germany; ^9^ Present address: Cardiovascular Department, the First Affiliated Hospital of Xi'an Jiaotong University 277 West Yanta Road Xi'an 710061 China

**Keywords:** Aldh9a1b, diabetic retinopathy, insulin resistance, omega‐6 fatty acids, prediabetes, trans, trans‐2,4‐decadienal, zebrafish

## Abstract

Omega‐6 fatty acids are the primary polyunsaturated fatty acids in most Western diets, while their role in diabetes remains controversial. Exposure of omega‐6 fatty acids to an oxidative environment results in the generation of a highly reactive carbonyl species known as trans, trans‐2,4‐decadienal (tt‐DDE). The timely and efficient detoxification of this metabolite, which has actions comparable to other reactive carbonyl species, such as 4‐hydroxynonenal, acrolein, acetaldehyde, and methylglyoxal, is essential for disease prevention. However, the detoxification mechanism for tt‐DDE remains elusive. In this study, the enzyme Aldh9a1b is identified as having a key role in the detoxification of tt‐DDE. Loss of Aldh9a1b increased tt‐DDE levels and resulted in an abnormal retinal vasculature and glucose intolerance in aldh9a1b^−/−^ zebrafish. Transcriptomic and metabolomic analyses revealed that tt‐DDE and aldh9a1b deficiency in larval and adult zebrafish induced insulin resistance and impaired glucose homeostasis. Moreover, alterations in hyaloid vasculature is induced by aldh9a1b knockout or by tt‐DDE treatment can be rescued by the insulin receptor sensitizers metformin and rosiglitazone. Collectively, these results demonstrated that tt‐DDE is the substrate of Aldh9a1b which causes microvascular damage and impaired glucose metabolism through insulin resistance.

## Graphical abstract

Omega‐6 fatty acids, prevalent in Western diets, are oxidized to form trans, trans‐2,4‐decadienal (tt‐DDE). This study reveals ALDH9a1b as a novel detoxifying system for tt‐DDE, confirmed by a CRISPR KO line. Analysis of transcriptome, metabolomics, and molecular experiments shows that *aldh9a1b* mutation not only alters tt‐DDE metabolism but induces diabetes and diabetic retinopathy, offering new insights into the omega‐6‐diabetes link.

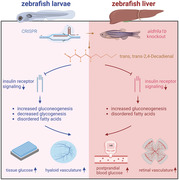

## 1 Introduction

Polyunsaturated fatty acids (PUFAs), primarily omega‐3 (n‐3) and omega‐6 (n‐6) fatty acids, are a group of essential nutrients that must be obtained through diet. A balanced n‐6/n‐3 ratio, ideally between 1:1 and 2:1, is crucial for preventing chronic diseases and maintaining overall health.^[^ ^1^ ^]^ Modern food processing, however, has shifted this ratio toward an unhealthy 15:1 to 16.7:1 in typical Western diets, with linoleic acid dominating over 90% of dietary PUFAs.^[^ ^1^, ^2^ ^]^ N‐3 fatty acids supplementation is advocated for its beneficial cardiovascular effects, whereas n‐6 fatty acids exhibit a complex relationship with cardiovascular disease (CVD). They are known to contribute to inflammatory responses, vascular constriction, and platelet aggregation, which are crucial in the onset of atherosclerosis and subsequent acute CVD incidents.^[^ ^3^, ^4^ ^]^ In contrast, n‐6 fatty acids are thought to reduce CVD risk by modulating serum lipid profiles and decreasing blood pressure.^[^ ^5^, ^6^, ^7^ ^]^ Linoleic acid, in particular, can be metabolized into cardioprotective agents like nitrated linoleic acid.^[^ ^8^ ^]^ However, there is a growing concern regarding the increased consumption of n‐6 PUFAs, especially when exposed to oxidative conditions like heating, which leads to the formation of highly reactive carbonyl species (RCS), mainly trans, trans‐2,4‐decadienal (tt‐DDE).^[^ ^9^, ^10^ ^]^ Comparable to other RCSs like 4‐hydroxynonenal (4‐HNE), acrolein (ACR), acetaldehyde, and methylglyoxal (MG), the timely and efficient detoxification of tt‐DDE is essential to prevent harm and associated diseases.^[^ ^11^ ^]^

RCS can be detoxified by enzyme systems, such as aldehyde dehydrogenases (Aldh), aldo‐keto reductases (Akr), and glyoxalase (Glo).^[^ ^11^, ^12^, ^13^ ^]^ The loss of these detoxifying enzymes created a specific signature of upregulated RCS, which can impact glucose homeostasis and cause microvascular complications through various pathways.^[^ ^12^, ^14^, ^15^, ^16^ ^]^ For example, MG, which is a precursor of advanced glycation end products (AGEs), leads to insulin resistance when combined with excessive calorie consumption, while it has relatively mild effects on glucose metabolism under a normal diet.^[^ ^16^ ^]^ Additionally, 4‐HNE disrupts pancreas development and results in alterations associated with type 1 diabetes (T1D), while acrolein, which belongs to Akr system, impaired insulin sensitivity, and glucose homeostasis similar to type 2 diabetes (T2D).^[^ ^15^, ^17^ ^]^ Given that RCS have marked effects on glucose homeostasis, identifying and targeting their specific detoxification pathways could be of great benefit in preventing, treating, and delaying the onset of diabetes. How levels of some of the RCS are regulated is known as Glo enzymes detoxify MG while Aldh3a1 detoxifies 4‐HNE. One enzyme of interest with respect to RCS is *aldh9a1b*, which is involved in lipid metabolism and was upregulated in *glo1* knockout zebrafish, suggesting its potential compensatory role in RCS detoxification in diabetes.^[^ ^16^, ^18^, ^19^, ^20^, ^21^, ^22^ ^]^

tt‐DDE, as an unsaturated aldehyde of medium‐polarity and medium acyl chain length, is mainly derived from the oxidation of the n‐6 fatty acids linoleic acid and arachidonic acid.^[^ ^9^, ^10^ ^]^ More specifically, tt‐DDE is generated during the decomposition of the linoleic acid derivative 9‐hydroperoxide,^[^ ^23^ ^]^ as well as of the arachidonic acid‐derivative 1‐hydroperoxyicosatetraenoic acid.^[^ ^24^ ^]^ A marked increase in the n‐6 PUFA content of the Western diet has resulted in tt‐DDE becoming the most abundant aldehyde in heated edible oils and cooking oil fumes, as well as being used as a food additive.^[^ ^9^, ^25^, ^26^, ^27^ ^]^ In vitro digestion model, tt‐DDE remained bioaccessible after digestion and potentially reached systemic circulation.^[^ ^28^ ^]^ Accumulated evidence suggests that it exerts adverse effects on human health, e.g., promotes the development of cardiovascular disease.^[^ ^29^, ^30^, ^31^, ^32^ ^]^ Comparative analysis of various unsaturated aldehydes, such as ACR, crotonaldehyde, trans‐2‐pentenal, trans, trans‐2,4‐heptadienal, tt‐DDE, and 4‐HNE, identified tt‐DDE as the most deleterious to human endothelial cells.^[^ ^30^ ^]^ There are also metabolic consequences of elevated tt‐DDE as Sprague‐Dawley rats fed a tt‐DDE‐rich diet developed fasting hyperglycemia, suggesting the high potential of tt‐DDE in the development of diabetes.^[^ ^31^ ^]^ Given this background it follows that approaches to detoxify tt‐DDE could be exploited to delay the development of diabetes in some conditions, e.g. obesity. Unfortunately, the route of tt‐DDE detoxification and the consequences of impaired detoxification, particularly in metabolic diseases such as diabetes and hyperlipidemia‐driven atherosclerosis, remain unknown.

Given the potential of *aldh9a1b* in RCS detoxification, we established an *aldh9a1b* knockout zebrafish line in order to investigate its role in glucose metabolism and related microvascular diseases. Our study identified tt‐DDE as a substrate for Aldh9a1b, and showed that its accumulation resulted in the pathological progression of diabetic retinopathy by inducing insulin resistance and impaired glucose homeostasis.

## 2 Results

### 2.1 Generation and Validation of Aldh9a1b−/‐ Zebrafish

**Figure 1:**
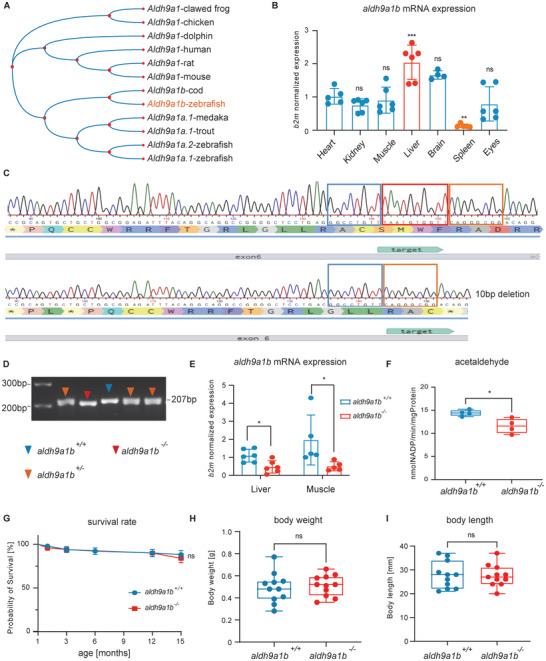
Generation and validation of *aldh9a1b^−/‐^* zebrafish line using CRISPR/Cas9 technology (A) Neighbor‐joining phylogenetic bootstrap tree of amino acid sequence showed the evolutionary relationship of *aldh9a1* and its isoforms in representative vertebrate animal species. B) *aldh9a1b* mRNA was mostly expressed in the liver, while least expressed in the spleen in *aldh9a1b^+/+^* adult zebrafish, *n* = 5/6. C) *aldh9a1b* CRISPR knockout line was designed to target exon 6 and successfully generated a 10 bp deletion validated in cDNA sequencing. The 10 bp deletion resulted in an artificial stop codon indicated with a star. D) *aldh9a1b^+/+^*, *aldh9a1b^+/−^*, *and aldh9a1b^−/−^* zebrafish can be distinguished on a genotyping‐PCR gel. The blue, red, and yellow arrows indicated PCR products of cDNA from *aldh9a1b^+/+^*, *aldh9a1b^+/−^*, *and aldh9a1b^−/−^* zebrafish. E) mRNA levels of *aldh9a1b* were significantly decreased in livers and muscles of *aldh9a1b^−/−^* mutants, *n* = 6/5. F) *aldh9a1b^−/−^* mutants showed decreased Aldh enzyme activity using the substrate acetaldehyde at 5dpf, *n* = 4, 120 larvae per clutch. G) Survival rate was not significantly changed between *aldh9a1b^+/+^* and *aldh9a1b^−/‐^* zebrafish over the age of 1–15 months, *n* = 51 and *n* = 53. H,I) Adult *aldh9a1b^−/−^* mutants displayed a normal body weight (H) and body length (I) compared to *aldh9a1b^+/+^* zebrafish, *n* = 11. mRNA Expression was quantified by RT‐qPCR and normalized to b2m. Each data point in this figure represented 20 larvae or one adult fish. The bars indicate mean ± SD values. Statistical analysis was performed by one‐way ANOVA, Student's *t*‐test, and log‐rank test. *ns* = not significant, ^*^ *p* < 0.05, ^**^ *p* < 0.01, ^***^ *p* < 0.001. b2m, 𝛽2 microglobulin; Bp, base pair; dpf, days post fertilization.

In zebrafish, three homologs for *aldh9a1*, including *aldh9a1a.1*, *aldh9a1a.2*, and *aldh9a1b*, have been identified. Phylogenetic analysis of the predicted Aldh9a1 amino acid sequences exhibited a close evolutionary relationship between Aldh9a1b and Aldh9a1(**Figure** **1**A). Furthermore, amino acid sequence alignment revealed that Aldh9a1b shared high similarity, including the same active and binding site with Aldh9a1 in both humans and mouse, indicating similar biological functions among them (Figure S1A, Supporting Information).

To investigate its role in zebrafish growth and development, the expression of *aldh9a1b* mRNA was determined. In the larval stages, *aldh9a1b* expression didn't show a significant alteration from 1dpf to 5dpf (Figure S1B, Supporting Information). In adult zebrafish, *aldh9a1b* mRNA was highest in the liver and lowest in the spleens (Figure 1B). These data suggested that *aldh9a1b* is distributed widely in different stages and organs and is crucial to zebrafish development and physiological function.

To assess the importance of Aldh9a1b, we established an *aldh9a1b^−/−^* zebrafish line using the CRISPR/Cas9 system to target exon 6 of the *aldh9a1b* gene in the *Tg(fli1:EGFP)* reporter line. Sequencing of the genomic DNA of *aldh9a1b^−/−^* zebrafish identified a frameshift mutation that was generated by a 10 bp deletion, which further resulted in an early stop codon in the amino acid sequence (Figure 1C). To confirm the successful generation of the *aldh9a1b^−/−^* zebrafish line, different validation experiments were performed on genomic DNA, mRNA, and different functional levels. *Aldh9a1b^+/+^*, *aldh9a1b^+/‐^* and *aldh9a1b^−/−^* zebrafish could be clearly distinguished on a genotyping‐PCR gel from genomic DNA (Figure 1D) and *aldh9a1b* mRNA levels were significantly decreased in the larvae, livers and muscles of *aldh9a1b^−/−^* mutants (Figure 1E; Figure S1C, Supporting Information). In addition, when total Aldh activity was measured using acetaldehyde as a substrate, there was a clear decrease in activity in *aldh9a1b^−/−^* larvae (Figure 1F). Subsequent analysis showed that loss of *aldh9a1b* did not affect the survival of adult zebrafish maintained under standard conditions for up to 15 months of age (Figure 1G). There were no noticeable alterations in the overall morphology of adult zebrafish or the body length or weight of the *aldh9a1b^−/−^* mutants (Figure 1H,I; Figure S1D, Supporting Information). Moreover, analysis of genotypes after heterozygous incrosses revealed the expected distribution equal to the Mendelian inheritance of *aldh9a1b* larvae (Figure S1E, Supporting Information). Together, the above data document the successful generation of *aldh9a1b* knockout zebrafish.

### 2.2 Alteration of the Retinal Vasculature in Aldh9a1b−/− Mutants

**Figure 2:**
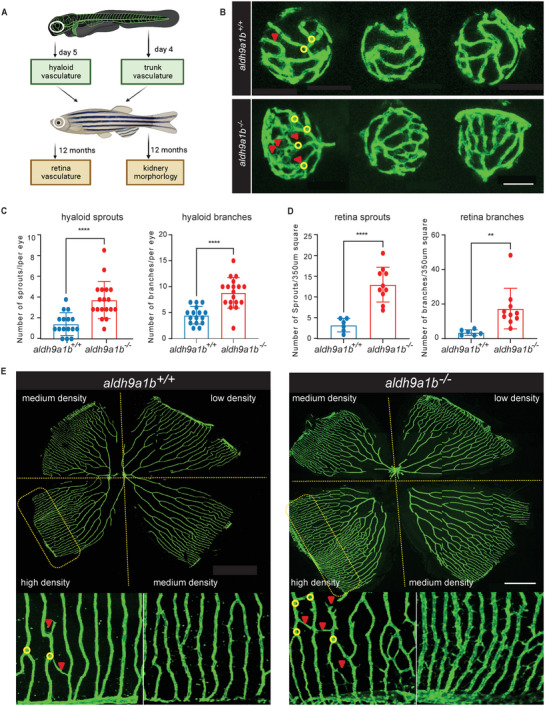
Alteration of the retinal vasculature in *aldh9a1b^−/−^* mutants (A) Graphical overview of phenotype exploration in *aldh9a1b^−/‐^* zebrafish. B) Representative confocal images of hyaloid vasculature showed vascular alterations in *aldh9a1b^−/‐^* larvae at 5dpf. Red arrows, sprouts; yellow circles, branch points. White scale bar = 50 µm. C) Quantification of increased hyaloid branchpoints and sprouts formation in *aldh9a1b^−/‐^* larvae, *n* = 16/18. D) Quantification of increased retinal branch points and sprouts formation in adult *aldh9a1b^−/‐^* zebrafish. One data point means one 350 µm^2^ square in high‐density retina, *n* = 8/10. E) Representative confocal images of adult retinal vasculature showed vascular alterations in *aldh9a1b^−/‐^* zebrafish. Red arrows, sprouts; yellow circles, branch points. White scale bar = 500 µm. The bars indicate mean ± SD values. Statistical analysis was performed by Student's *t*‐test, ^**^ *p* < 0.01, ^****^ *p* < 0.0001.

Diabetic nephropathy and diabetic retinopathy are common microvascular complications that severely compromise the quality and life expectancy of diabetic patients.^[^ ^33^ ^]^ Therefore, we studied potential microvascular complications of *aldh9a1b* knockout in larval and adult *aldh9a1b^−/−^* zebrafish (**Figure** **2**A). In larvae, the trunk vasculature and the hyaloid vasculature were analyzed. We found increasing numbers of hyaloid sprouts and branches in *aldh9a1b^−/^* ^−^ eyes, while trunk vasculature didn't show altered structures compared to *aldh9a1b^+/+^* larvae (Figure 2B,C; Figure S2A,B, Supporting Information). In adult zebrafish, the retinal vasculature and kidney morphology were examined by confocal and electron microscopy. *aldh9a1b^−/−^* zebrafish displayed significantly increasing sprouts and branches in the retinal vessels, while in the kidney, gross morphology and glomerular basement membrane (GBM) thickness identified by PAS staining or electron microscope remained unaltered (Figure 2D,E; Figure S2C–E, Supporting Information). Thus, *aldh9a1b^−/−^* mutant zebrafish show incipient hallmarks of diabetic retinopathy in the zebrafish larvae as well as in adults.

### 2.3 Link Between tt‐DDE, Aldh9a1b, and Angiogenesis

**Figure 3:**
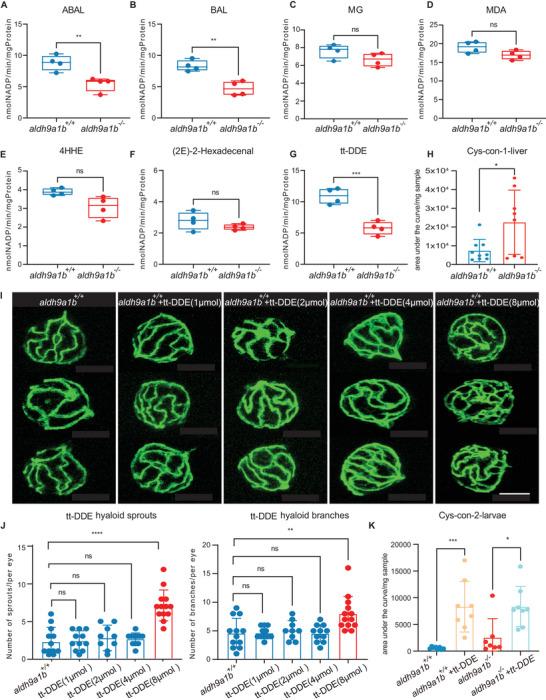
The link between tt‐DDE, Aldh9a1b, and angiogenesis (A‐G) Aldh enzyme activity was significantly decreased using substrate ABAL (A), BAL (B), and tt‐DDE (G), but unaltered with substrate MG (C), MDA (D), 4HHE (E) and 2(E)−2‐hexadecenal (F) in *aldh9a1b^−/‐^* larvae at 5dpf. Each data point represented 120 larvae per clutch, *n* = 4. H) Cys‐con‐1 was significantly changed in *aldh9a1b^−/−^* livers. *n* = 9/8. I) Representative confocal images of hyaloid vasculature showed vascular alterations in *aldh9a1b^+/+^* larvae treated with 0–8 µmol tt‐DDE at 5dpf. White scale bar = 50 µm. J) Quantification of increased hyaloid branch points and sprouts formation in 8 µmol tt‐DDE treated *aldh9a1b^+/+^* zebrafish larvae, while no significant change in 1, 2, and 4 µmol tt‐DDE treatments were observed. One data point means one hyaloid per larva. K) Cys‐con2 was significantly increased in tt‐DDE‐treated larvae. *n* = 8/7. The bars indicate mean ± SD values. Statistical analysis was performed by Student's *t*‐test and one‐way ANOVA. *ns* = not significant, ^*^ *p* < 0.05, ^**^ *p* < 0.01, ^***^ *p* < 0.001, ^****^ *p* < 0.0001.

Aldh enzymes catalyze the oxidation and inactivation of a wide spectrum of aldehydes, and their dysfunction results in the accumulation of toxic aldehydes and lead to severe diseases.^[^ ^34^ ^]^ Since Aldh9a1b substrates had not studied previously, several potential substrates for Aldh9a1 were analyzed in *aldh9a1b* mutants^[^ ^35^, ^36^ ^]^ (Figure S3, Supporting Information). Betaine aldehyde (BAL) and 4‐aminobutyraldehyde (ABAL) were the potentially most likely candidates based on their kinetic parameters for Aldh9a1.^[^ ^35^, ^36^ ^]^ However, since MG, malondialdehyde (MDA), 4‐hydroxyhexenal (4‐HHE), (2E)−2‐hexadecenal and tt‐DDE were all RCS with the potential to target the vasculature, they were also included in the study.^[^ ^14^, ^31^, ^37^, ^38^, ^39^ ^]^ The lack of Aldh9a1b had no impact on the metabolism of MG, MDA, 4‐HHE and 2(E)−2‐hexadecenal but significantly decreased the generation of NADP in samples treated with ABAL, BAL, and tt‐DDE (**Figure** **3**A–G). Importantly, substrate whose metabolism was most affected in the *aldh9a1b^−/−^* mutants was tt‐DDE. tt‐DDE is reported to be metabolized to 2,4‐decadienoic acid and cysteine‐conjugated 2,4‐decen‐1‐ol (Cys‐con) by virtue of ALDH enzymatic activity and glutathione (GSH) conjugation respectively^[^ ^40^ ^]^ (Figure S4A, Supporting Information). To validate the impaired detoxification of tt‐DDE, six Cys‐con metabolites were identified during the HPLC/MS analyses, which not only shared the same mass‐to‐charge ratio, but also the same targeted fragmentation pattern (Figure S4A,B, Supporting Information). Differences between these six metabolites were based on the retention time, hence the occurrence of isomers/diastereomers is the most logical explanation. Since Cys‐con was biomarker of tt‐DDE exposure and *aldh9a1b* knockout increases Cys‐con significantly in liver and muscle samples, the impaired detoxification of tt‐DDE induced by *aldh9a1b* knockout was validated (Figure 3H; Figure S4, Supporting Information). Moreover, GSH conjugated tt‐DDE also increased in *aldh9a1b^−/−^* livers (Figure S4E, Supporting Information).

In order to verify that tt‐DDE is a substrate for Aldh9a1b, incubation experiments in *aldh9a1b^+/+^* zebrafish were treated with different concentrations of tt‐DDE (Figure S5, Supporting Information). The effects were compared with those of ABAL (Figure S6, Supporting Information) and BAL (Figure S7, Supporting Information). 20 µmol tt‐DDE caused a significant lethality and a lower concentration (10 µmol) elicited significant morphological changes (Figure S5A–D, Supporting Information). Therefore, concentrations of up to 8 µmol tt‐DDE were considered as safe dose for zebrafish. Subsequently, analysis and quantification of the hyaloid vasculature identified increased hyaloid branchpoints and sprout formation in tt‐DDE treated larvae similar to the microvascular phenotype observed in *aldh9a1b^−/−^* larvae (Figure 3I,J). In contrast, treatment with neither ABAL nor BAL was able to generate comparable alterations in the hyaloid vasculature of zebrafish larvae (Figures S6–S7, Supporting Information). Furthermore, elevated concentration of Cys‐con was detected in tt‐DDE‐treated larvae, confirming a successful establishment of tt‐DDE exposure model (Figure 3K). These findings go a long way to linking the metabolism of tt‐DDE by Aldh9a1b with vascular alterations in zebrafish.

### 2.4 Downregulated Insulin Receptor Signaling Pathway in *Aldh9a1b^−/‐^* Mutants and tt‐DDE Treated Zebrafish

**Figure 4:**
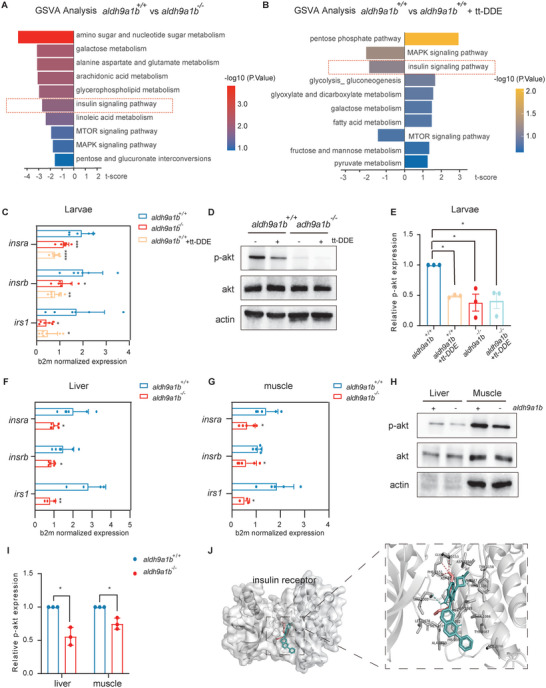
Downregulated insulin receptor signaling pathway in *aldh9a1b^−/‐^* and in tt‐DDE treated zebrafish (A) GSVA analysis of RNA‐seq displayed top 10 insulin receptor signaling and carbon metabolism‐related pathways in *aldh9a1b^−/−^* larvae compared to *aldh9a1b^+/+^* larvae at 5 dpf. Left, downregulated pathways; right; upregulated pathways. B) GSVA analysis of RNA‐seq displayed top 10 insulin receptor signaling and carbon metabolism‐related pathways in tt‐DDE treated *aldh9a1b^+/+^* larvae compared to *aldh9a1b^+/+^* controls. Left, downregulated pathways; right; upregulated pathways. C) mRNA expression of *insra*, *insrb*, and *irs1* were significantly decreased in *aldh9a1b^−/−^* larvae and tt‐DDE treated *aldh9a1b^+/+^* larvae at 5dpf, *n* = 6/7. D,E) Representative Western blot (D) and quantification (E) showed decreased p‐Akt activation in *aldh9a1b^−/−^* larvae and tt‐DDE treated *aldh9a1b^+/+^* larvae at 5dpf. Total Akt protein served as a loading control, *n* = 3. F,G) mRNA expression of *insra*, *insrb*, and *irs1* were significantly decreased in livers (F) and muscles (G) of *aldh9a1b^−/−^* adult zebrafish, *n* = 6/5. H,I) Representative Western blot and quantification showed reduced *p*‐Akt phosphorylation in *aldh9a1b^−/−^* in livers and muscles. J) Docking analysis showed tt‐DDE shared same binding pocket with linsitinib and bound to PHE1151, GLY1152 and MET1153 of insulin receptor. Pink sticks = tt‐DDE, blue sticks = linsitinib, dash line = hydrogen bonds. Total Akt served as loading control, *n* = 3. mRNA Expression was quantified by RT‐qPCR and normalized to b2m. Each data point in this figure represented 20 larvae or one organ per fish. The bars indicate mean ± SD values. Statistical analysis was performed by Student's *t*‐test and one‐way ANOVA, ^*^ *p* < 0.05, ^**^ *p* < 0.01, ^***^ *p* < 0.001, ^****^ *p* < 0.0001.

To investigate potential links between Aldh9a1b and metabolism, *aldh9a1b^−/−^* and *aldh9a1b^+/+^* zebrafish larvae as well as *aldh9a1b^+/+^* zebrafish treated with tt‐DDE were subjected to RNA‐sequencing. Principal component analysis (PCA) demonstrated that the samples within each group clustered separately from the other groups (Figure S8A, Supporting Information). Overall, this approach identified 940 differentially regulated genes in *aldh9a1b^−/−^* versus *aldh9a1b^+/+^* zebrafish and 239 differential genes in the tt‐DDE versus control *aldh9a1b^+/+^* group. Pathway enrichment analysis using the Kyoto Encyclopedia of Genes and Genomes (KEGG) database identified significant changes in pathways involving arachidonic acid and linoleic acid metabolism (Figure S8B,C, Supporting Information),^[^ ^41^ ^]^ which reflects the fact that n−6 PUFAs are the main source of tt‐DDE. In the tt‐DDE treated group, insulin signaling and related pathways accounted for over half of the altered pathways (Figure S8D,E, Supporting Information). Subsequent gene set variation analysis (GSVA) identified the 10 pathways most altered by the metabolite as carbon metabolism and insulin signaling (**Figure** **4**A,B). Only the change of insulin signaling and its downstream pathways (MAPK and MTOR signaling) were consistent in both groups, which suggested an altered insulin signaling as an inducer of vascular alterations in tt‐DDE treated and in *aldh9a1b^−/−^* zebrafish.

To validate the RNA‐sequencing results, the expression and activity of insulin signaling molecules including *insulin* (*ins*), *insulin receptor* (*insr*), *insulin receptor substrate* (*irs*), and *akt* were quantified by qPCR and Western blot. mRNA expression of *insulin* showed a significant reduction in the tt‐DDE treated zebrafish, while it remained unchanged in *aldh9a1b^−/−^* zebrafish (Figure S9A, Supporting Information). Downregulated insulin expression in tt‐DDE treated larvae suggested tt‐DDE may harm the development of the pancreas, but *pdx1* (*pancreatic and duodenal homeobox 1*), which is essential for pancreatic development, was not changed significantly (Figure S9B, Supporting Information). The expression of additional components of the signaling pathway, i.e., *insra*, *insrb* and *irs1* showed a similar decline in both *aldh9a1b^−/−^* zebrafish and in tt‐DDE treated larvae (Figure 4C). Akt phosphorylation, which is an essential downstream event in the insulin signaling, also strongly decreased in both groups (Figure 4D,E). As liver and muscle are the two most important target organs for insulin and *aldh9a1b* expression was highest in the liver, insulin signaling was also examined in these organs. While *ins* mRNA levels were comparable in all three groups, again the expression of *insra*, *insrb* and *irs1* and the phosphorylation of Akt were decreased in the absence of *aldh9a1b* and in the presence of tt‐DDE (Figure 4F–I; Figure S9C,D, Supporting Information). Lastly, *nos2b*, which is considered to act as a homologue of human endothelial nitric oxide synthase (eNOS) and regulates endothelial function, was also decreased in *aldh9a1b^−/−^* zebrafish and tt‐DDE treated larvae^[^ ^42^ ^]^ (Figure S9E–G, Supporting Information). Expression of vascular endothelial growth factor was not significantly altered in *aldh9a1b^−/−^* mutants (Figure S9H, Supporting Information).

To investigate a potential interaction between tt‐DDE and the insulin receptor, we performed docking analysis using AutoDock Vina. Linsitinib (OSI‐906), a classic nonpeptide small molecule, inhibits the tyrosine kinase activity of the insulin receptor and was used as a positive control in this analysis.^[^ ^43^ ^]^ The docking model revealed that tt‐DDE shared same binding pocket with linsitinib. The binding is characterized by a relatively low binding energy of −4.8 kcal/mol and formed three visible hydrogen bonds with PHE1151, GLY1152, and MET1153, indicating a highly stable binding between tt‐DDE and the insulin receptor (Figure 4J). Collectively, the data described above suggest that in the absence of Aldh9a1b or in the presence of tt‐DDE the insulin signaling pathway is dysfunctional, providing an intrinsic explanation for the observed microvascular alteration in the retina.

### 2.5 Impaired Glucose Homeostasis in *Aldh9a1b^−/‐^* and tt‐DDE Treated Zebrafish

**Figure 5:**
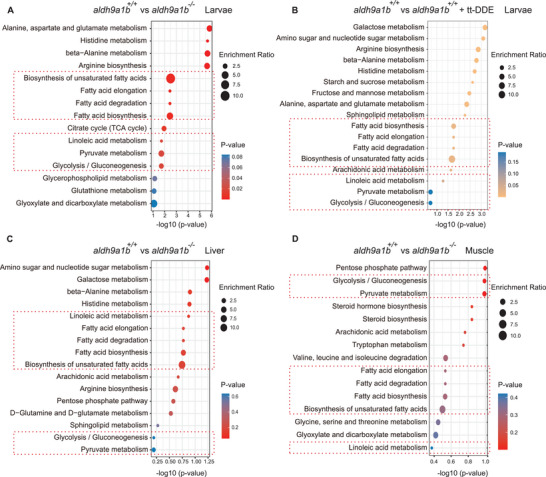
Altered glucose metabolism in *aldh9a1b^−/‐^* and tt‐DDE treated zebrafish (A) Enrichment analysis of targeted metabolomics displayed top carbon metabolic pathways in *aldh9a1b^−/−^* larvae compared to *aldh9a1b^+/+^* larvae at 5 dpf. B) Enrichment analysis of targeted metabolomics showed top carbon metabolic pathways in tt‐DDE treated *aldh9a1b^+/+^* larvae compared to *aldh9a1b^+/+^* controls. C,D) Enrichment analysis of targeted metabolomics displayed top carbon metabolic pathways in *aldh9a1b^−/−^* livers (C) and muscles (D) compared to *aldh9a1b^+/+^*. The red frame represented overlapped altered pathways among four comparisons.

**Figure 6:**
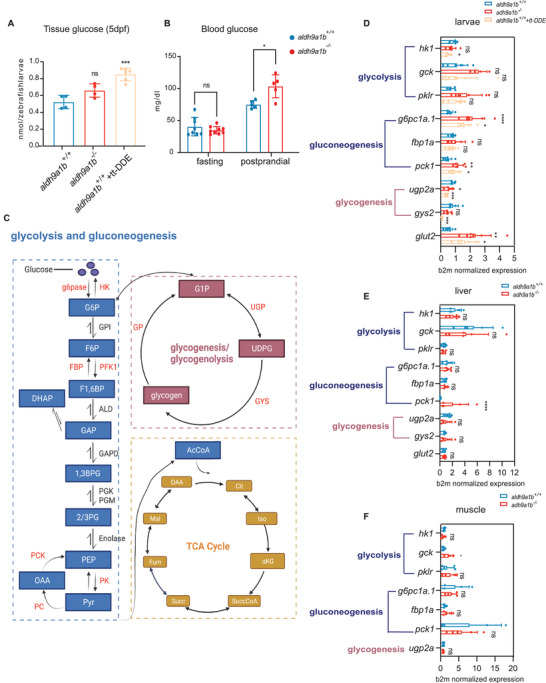
Impaired glucose homeostasis in *aldh9a1b^−/‐^* and tt‐DDE treated zebrafish (A) The whole‐body glucose was significantly increased in tt‐DDE treated *aldh9a1b^+/+^* larvae at 5 dpf. In *aldh9a1b^−/‐^* larvae, whole‐body glucose was also increased, but not significantly, *n* = 4/6. B) Blood glucose of *aldh9a1b^−/−^* adults did not show any alteration in fasting state but was significantly increased in the 2‐hour postprandial state. C) Graphical overview of key biological processes in glucose metabolism. D) mRNA levels of key enzymes in glucose metabolism showed alterations of glycolysis, gluconeogenesis, and glycogenesis in *aldh9a1b^−/−^* and in tt‐DDE treated *aldh9a1b^+/+^* larvae at 5dpf, *n* = 6/7. E,F) mRNA levels of key enzymes in glucose metabolism showed alterations of glycolysis, gluconeogenesis and glycogenesis in the livers (E) and muscles (F) of *aldh9a1b^−/−^* zebrafish, *n* = 6/5. mRNA Expression was quantified by RT‐qPCR and normalized to b2m. Each data point in this figure represented 20 larvae or one fish. The bars indicate mean ± SD values. Statistical analysis was performed by Student's *t*‐test and one‐way ANOVA, ^*^ *p* < 0.05, ^**^ *p* < 0.01, ^***^ *p* < 0.001, ^****^ *p* < 0.0001.

To determine the exact downstream insulin signaling induced by *aldh9a1b^−/−^* and tt‐DDE, glucose‐related metabolites, such as fatty acids and carbohydrates, were screened by GC/MS in larvae and adult zebrafish organs. Partial least squares‐discriminant analysis (PLS‐DA) exhibited separated groups by comparing their component composition, indicating significant differences among different groups (Figure S10A–C, Supporting Information). Thus, fold change analysis was performed to determine differential metabolites. Almost half of all the analyzed metabolites in the larvae, livers and muscles of adult zebrafish were significantly changed due to *aldh9a1b* loss and tt‐DDE treatment (Figure S10D–G, Supporting Information). Moreover, enrichment analysis of screened metabolites displayed overlapped pathways directly connected to fatty acids metabolism and glucose metabolism among the four groups, which not only resemble the results of RNA‐seq but also strengthen that *aldh9a1b^−/‐^* and tt‐DDE altered glucose metabolism (**Figure** **5**A–D). Therefore, the glucose level was determined in larvae and adult zebrafish to investigate how *aldh9a1b^−/‐^* and tt‐DDE influence glucose metabolism. The whole‐body glucose in larvae showed an increasing trend in *aldh9a1b^−/‐^* mutants and in tt‐DDE treated animals, but was only significantly increased in tt‐DDE treated larvae (**Figure** **6**A). Yet, in adult *aldh9a1b^−/‐^* zebrafish increased postprandial blood glucose was observed, while in overnight‐fasted *aldh9a1b^−/‐^* zebrafish blood glucose remained unaltered, demonstrating impaired glucose homeostasis in adult *aldh9a1b^−/‐^* zebrafish and in tt‐DDE treated larvae (Figure 6B).

To further analyze the glucose metabolism in *aldh9a1b^−/‐^* zebrafish, the expression of key enzymes of the glucose metabolism was investigated. The rate‐limiting enzymes for glycolysis are *hexokinase 1* (*hk1*), *glucokinase* (*gck*, also termed *hk4*) and *pyruvate kinase L/R* (*pklr*); for gluconeogenesis are *glucose‐6‐phosphatase catalytic subunit 1a* (*g6pc1a.1*), *fructose‐1,6‐bisphosphatase 1a* (*fbp1a*) and *phosphoenolpyruvate carboxykinase 1* (*pck1*); for glycogenesis are *UDP‐glucose pyrophosphorylase 2a* (*ugp2a*) and *glycogen synthase 2* (*gys2*) (Figure 6C). *aldh9a1b^−/−^* larvae displayed a significant increasing expression of *g6pc1a.1* and *pck1*, while decreasing expression of *ugp2a*, representing upregulated gluconeogenesis and downregulated glycogenesis. Furthermore, the data revealed increasing glucose production and decreasing glucose utilization, thus explaining the hyperglycemia in *aldh9a1b^−/‐^* mutants and in tt‐DDE treated larvae (Figure 6D). Lastly, glucose transporter 2 (GLUT2), an insulin‐independent glucose transporter, significantly increased as possible feedback to hyperglycemia in *aldh9a1b^−/−^* zebrafish and in the tt‐DDE group (Figure 6D). In adult *aldh9a1b^−/‐^* zebrafish, the liver, as the most important glucose regulatory organ, displayed similarly changed gluconeogenesis as seen in larvae (Figure 6E), while in muscles, glycolysis was significantly altered (Figure 6F).

### 2.6 Altered Angiogenic Hyaloid Vasculature Caused by *Aldh9a1b* Knockout and tt‐DDE Treatment Can be Rescued by Metformin, Rosiglitazone, and Carnosine

**Figure 7:**
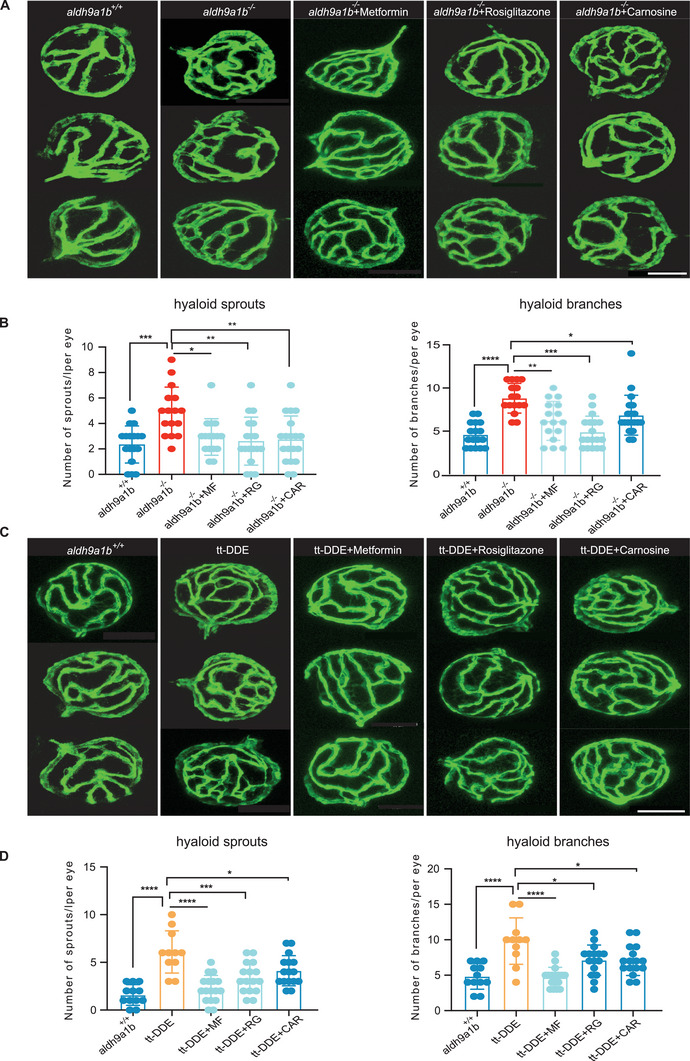
Altered angiogenic hyaloid vasculature caused by *aldh9a1b* knockout and tt‐DDE treatment can be rescued by metformin, rosiglitazone and carnosine (A) Representative confocal images of hyaloid vasculature showed vascular alterations and beneficial effects in *aldh9a1b^−/−^* zebrafish larvae treated with MF, RG, and CAR. White scale bar = 50 µm. B) Quantification of hyaloid branch points and sprouts formation showed increased angiogenic vasculature in *aldh9a1b^−/‐^* can be rescued by metformin, rosiglitazone and carnosine. One data point means one hyaloid per larva. C) Representative confocal images of hyaloid vasculature showed vascular alterations and beneficial effects in *aldh9a1b^+/+^* larvae co‐incubated with tt‐DDE and MF, RG, and CAR. White scale bar = 50 µm. D) Quantification of hyaloid branch points and sprout formation showed that increased angiogenic vasculature induced by tt‐DDE can be rescued by metformin, rosiglitazone, and carnosine. One data point means one hyaloid per larva. The bars indicate mean ± SD values. Statistical analysis was performed by one‐way ANOVA. ^*^ *p* < 0.05, ^**^ *p* < 0.01, ^***^ *p* < 0.001, ^****^ *p* < 0.0001. MF, metformin; RG, rosiglitazone; CAR, carnosine; PK, PK11195.

All the above data have demonstrated that downregulated insulin signaling and impaired glucose metabolism could be responsible for the angiogenic retinal vasculature in *aldh9a1b^−/−^* zebrafish and in tt‐DDE treated animals. To validate the results further, several anti‐diabetic drugs were used to perform rescue experiments. Metformin and rosiglitazone are first‐line insulin receptor sensitizers in type 2 diabetes. Besides, previous studies suggested 10 µmol metformin and 1 µmol rosiglitazone could rescue hyperglycemia in zebrafish.^[^ ^44^, ^45^, ^46^ ^]^ In our study, 10 µmol metformin and 1 µmol rosiglitazone also caused strong improvement of numbers of sprouts and branches in the hyaloid vasculature (Figures S11‐S12, Supporting Information). Therefore, we incubated *aldh9a1b^−/−^* larvae and tt‐DDE treated *aldh9a1b^+/+^* zebrafish larvae with 10 µmol metformin, 1 µmol rosiglitazone, and 10 mmol RCS scavenger–L‐carnosine. In *aldh9a1b^−/‐^* zebrafish, the altered hyaloid alterations were successfully reversed by metformin, rosiglitazone, and carnosine, while metformin and rosiglitazone provided the strongest rescue effects. (**Figure** **7**A–B). In tt‐DDE treated larvae, abnormal hyaloid vasculature could also be reversed by metformin, rosiglitazone and carnosine, while metformin worked most effectively among them on the whole (Figure 7C,D). In conclusion, the successful rescue effects of the anti‐hyperglycemic drugs validated further that the tt‐DDE phenotype involving insulin resistance, mild postprandial hyperglycemia and sustained alteration of lipid metabolisms resulted in the microvascular phenotype observed.

## 3 Discussion

In this study, we identified a novel contributor, tt‐DDE, to hyperglycemia, insulin resistance, and microvascular disease. As a common RCS in diet, the characterization and understanding of its detoxification process is crucial.^[^ ^47^, ^48^ ^]^ Previous reports have suggested tt‐DDE's potential detoxification through two classic pathways common to other α,β‐unsaturated aldehydes: GSH conjugation and aldehyde oxidation.^[^ ^40^, ^49^ ^]^ Thus, cysteine‐conjugated 2,4‐decadien‐1‐ol and GSH conjugated tt‐DDE were characterized, although the exact pathway how ALDHs are involved remains to be determined.^[^ ^40^ ^]^ Our data established an *aldh9a1b* knockout zebrafish model, and by utilizing this model, we have observed a reduction in ALDH enzyme activity in relation to tt‐DDE. Further, an increased level of tt‐DDE exposure, and overlapped phenotypic and molecular alterations resulting from *aldh9a1b* knockout and in vitro tt‐DDE exposure were detected. This is the first study to recognize Aldh9a1b as a detoxification system for tt‐DDE.

A recent study reported a trend of increased fasting blood glucose levels associated with a diet rich in tt‐DDE.^[^ ^31^ ^]^ Our findings mirror this pattern of increased fasting blood glucose, but we additionally discovered a significant increase in 2‐hour postprandial glucose and impaired insulin sensitivity by a combination of transcriptome and metabolomics analysis. Based on our own and other data, different RCS, including 4‐HNE, MDA, ACR and acetaldehyde have distinct effects in vivo and in the context of diabetes.^[^ ^14^, ^15^, ^17^, ^50^, ^51^, ^52^, ^53^ ^]^ With the addition of the recently discovered tt‐DDE as a new RCS that influences glucose homeostasis, we further expand our comprehension of how a specific RCS profile can result in altered glucose regulation, the onset of diabetes, and related organ complications.^[^ ^14^, ^15^, ^17^ ^]^

Moreover, tt‐DDE has been attributed with a pivotal role in cardiovascular diseases such as endothelial dysfunction, atherosclerosis, and hypertension, with dysregulation of eNOS being a key feature.^[^ ^30^, ^31^ ^]^ The reduction of eNOS also was referred as one of the major cause of vascular complications and induced by insulin resistance in diabetes.^[^ ^54^, ^55^ ^]^ Our data revealed that *nos2b*, an eNOS homolog, was also decreased by tt‐DDE exposure, indicating a modification to the downstream insulin signaling pathway. Furthermore, tt‐DDE, recognized as a reactive Michael acceptor, can modify proteins and DNAs such as cytochrome c, apo‐SOD1, and dGuo.^[^ ^56^, ^57^, ^58^, ^59^, ^60^, ^61^, ^62^, ^63^ ^]^ This modification process can lead to DNA damage, oxidative stress, and mitochondrial dysfunction, ultimately resulting in disordered energy metabolism. In contrast, docking analyses performed in our study suggests a high probability that tt‐DDE forms a stable bond with the insulin receptor via three hydrogen bonds, offering a different perspective of direct receptor modification by tt‐DDE. However, this does not rule out the potential of a Michael adduct between tt‐DDE and the insulin receptor. A comprehensive understanding of these mechanisms and the enhancement of future research into protein or DNA modifications, both in vivo and in vitro, necessitates the development of a tt‐DDE probe.

Human exposure to tt‐DDE is influenced by various factors including cooking methods, types of cooking oils, and food types.^[^ ^9^, ^26^ ^]^ Notably, tt‐DDE is predominantly found in plant‐derived oils, and it significantly contributes to the aroma of fried foods.^[^ ^26^ ^]^ For instance, heated sunflower oil was found to exhibit the highest tt‐DDE concentration, ranging between 29–129 µg/g. Furthermore, during the frying process, the atmospheric tt‐DDE concentrations fluctuated from 210–890 µg m^−^^3^ for potato chips and 476–698 µg m^−^^3^ for pork.^[^ ^27^, ^64^, ^65^, ^66^ ^]^ These findings suggested that human exposure to tt‐DDE was through both ingestion and inhalation, an exposure pattern that our zebrafish model mirrored. However, the absence of evidence for tt‐DDE measurement in cells or tissues currently posed a significant challenge in obtaining real human exposure data for a comprehensive comparison with our zebrafish model. In response to this limitation, we have developed a method to measure tt‐DDE exposure in tissues, revealing a quantifiable correlation between tt‐DDE exposure and insulin resistance. This approach offers a firm foundation for future investigations. Moreover, considering the documented harm caused by tt‐DDE to workers in specific occupations and the lack of clear legal guidelines regulating tt‐DDE concentrations in cooking oil fumes, our study serves as compelling evidence advocating for the establishment of tt‐DDE regulations and the monitoring of occupational exposure.

Lastly, the potentially harmful effects of a high intake of n‐6 fatty acids on diabetic patients remains a contentious issue.^[^ ^67^, ^68^, ^69^, ^70^ ^]^ In the United States, a dramatic 1000‐fold increase in vegetable oil consumption, predominantly rich in n‐6 fatty acids, coincides with a 12‐fold and 3‐fold rise in the prevalence of diagnosed diabetes and obesity, underscoring the critical need to understand the specific effects of n‐6 fatty acids on these conditions.^[^ ^71^, ^72^, ^73^ ^]^ Results from pooled analysis showed that the intake of linoleic acid was associated with a lower risk of T2D in a dose‐dependent manner.^[^ ^74^, ^75^ ^]^ However, another meta‐analysis posited that n‐6 PUFAs might act more as markers of hyperinsulinemia and obesity inducer than as protective factors for T2D.^[^ ^76^, ^77^ ^]^ The most extensive systematic review to date reported inconclusive effects of n‐6 fatty acids on the incidence of diabetes, due to the low quality of the evidence available.^[^ ^67^ ^]^ Our research sheds new light on the potential adverse effects of n‐6 fatty acid supplementation and its correlation with T2D, underscoring the necessity for careful consideration when implementing nutritional supplementation strategies. Considering polymorphism in aldehyde dehydrogenase gene superfamily,^[^ ^78^ ^]^ tt‐DDE might act as a significant inducer for specific populations, suggesting an individualized approach to n‐6 intake and T2D prevention strategy.

In conclusion, the zebrafish model has been shown to be useful to provide a previously unexpected explanation for the microvascular disorders seen in patients with prediabetes. Our data point to an important role of Aldh9a1b for controlling n‐6‐derived reactive RCS, especially tt‐DDE, which will if not sufficiently detoxified, trigger a cascade from insulin resistance, increased gluconeogenesis and altered fatty acid metabolism, consequently leading to microvascular disease.

## 4 Experimental Section

### Study Approval

All experimental procedures on animals were approved by the local government authority Regierungsprasidium–Karlsruhe and by Medical Faculty Mannheim (license no: G‐98/15 and I‐19/02) and carried out in accordance with the approved guidelines.

### Zebrafish Husbandry

The zebrafish line *Tg(fli1: EGFP)* was used and raised as described under a standard husbandry environment.^[^ ^79^, ^80^ ^]^ Embryos and larvae until 5dpf was held and raised in egg water at 28.5 °C with or without 0.003% 1‐phenyl‐2‐thiourea (PTU) (Sigma) to suppress pigmentation. Larvae older than 5dpf and adult fish were kept under a 13 h light/11 h dark cycle and were fed twice a day, freshly hatched Artemia Salina in the morning and fish flake food in the afternoon.

### Mutant Generation

The *aldh9a1b* knockout CRISPR zebrafish line was established as previously described.^[^ ^15^ ^]^ Briefly, guide RNA (gRNA) to target *aldh9a1b* exon 6 were designed with ZiFiT Targeter 4.2 and cloned into a T7‐driven promoter expression vector (pT7‐gRNA; Addgene). The pT3TSnCas9n vector (Addgene) was used to transcript in vitro and synthesize Cas9 mRNA after linearizing with xbal (Biolab).^[^ ^81^ ^]^ The T7 mMessage mMachine Kit (Invitrogen) and T3 MEGAshortscript (Invitrogen) were used to get gRNA and Cas9 mRNA respectively according to the suggestions of the manufacturer. At one‐cell stage, one nanoliter of 0.1 m KCl solution mixed with the gRNA (200 pg nL^−1^) and Cas9 mRNA (200 pg nL^−1^) was injected into the single‐cell stadium of an embryo. Generated F0 fish were analyzed for germline transmission and crossed with *Tg(fli1: EGFP)*. Sanger sequencing and gel electrophoretic separation of PCR products were used for genotyping. The genome and amino acid sequence were analyzed with benchling (benchiling.com).^[^ ^82^ ^]^

### Reverse‐Transcription Quantitative Polymerase Chain Reaction Analysis (RT‐qPCR)

Twenty larvae at 5dpf or one organ per adult fish were collected as one sample. Total RNA was extracted with the RNeasy Mini Kit (Qiagen) according to the manufacturer's protocol. 1 µg RNA was used to synthesize cDNA with the Maxima First Strand cDNA Synthesis Kit (Thermo Fisher Scientific), following the manufacturer's protocol. Primers were designed with NCBI primer blast and listed in Table S1 (Supporting Information). RT‐qPCR was done with Power SYBR Green PCR Master Mix Kit (Thermo Fisher Scientific) in 96 or 384 reaction plates and run by QuantStudio 3 or QuantStudio 5 Real‐Time PCR System (Thermo Fisher Scientific).

### Microscopy and Analysis of Vascular Alterations in Larvae and Adults

For imaging of zebrafish trunk vasculature in vivo, *aldh9a1b* larvae were incubated with PTU from day 1. At 4 dpf, larvae were anesthetized in 0.0003% tricaine and put in 96 well plates, one larva in one well. Images were acquired by a confocal microscope (DM6000 B) with a Leica TCS SP5 DS scanner, set 1024 × 1024 pixels, 0.8 µm Z‐steps and 20 × 0.7 objective. Ten pairs of trunk vasculature were observed followed by the first 5 ISVs. Small newly developed vessels were referred to as “hyperbranched”, while malformed vessels (wrong direction, without connection, too thin or too thick) were regarded as abnormal ISVs

For imaging of zebrafish hyaloid vasculature in vivo, *aldh9a1b* larvae were put in 0.003% tricaine and fixed in 4% PFA overnight at 5dpf. Fixed larvae were washed for 10 min three times with double distilled water (ddH_2_O) and incubated in 0.3% Trypsin/EDTA solution (Gibco) buffered with TRIS HCl (1.5 m, pH 7.8) for 90 min at 37 °C. After being washed three times, larvae were dissected to get hyaloid and put in PBS for visualization.^[^ ^83^ ^]^ The confocal microscope was described above with a setting of 1024 × 1024 pixels, 0.6 µm Z‐steps, and 20 × 3 objectives.

For imaging of zebrafish retina vasculature in vivo, the method and analysis were described as a previous study.^[^ ^84^ ^]^ Briefly, PFA‐fixed heads from adult zebrafish were put on an agarose platform covered with cold 1x PBS. The retina was detached from the eyes and washed three times. Afterward, it was transferred to a slide, immersed in mounting media, and covered with a cover slide. The confocal microscope was described above with a setting of 1024 × 1024 pixels, 1.5 µm Z‐steps, and 20 × 1 objective. GIMP and ImageJ were used to quantify the sprouts and branches in squares of 350 × 350 µm^2^.

### Analysis of Kidney Morphology

For Periodic acid–Schiff (PAS) staining, kidneys were detached from adult zebrafish and fixed in 4% PFA. Fixed kidneys were embedded in paraffin and cut into 4 µm thick sections with a Leica RM2235 microtome and placed on a slide. After deparaffinization, sections were immersed in 1% periodic acid for 10 min, washed by ddh_2_O three times, and put in Schiff's reagent for 20 min, then in SO_2_ water for 2 min three times. Afterward, sections were washed with running tap water and stained in hematoxylin solution. Stained sections were dehydrated with ethanol solutions and mounted with a mounting medium. Brightfield imaging was pictured with a scanner (Zeiss Axio Scan.Z1). Analysis of glomerular structure was performed as recently described.^[^ ^38^ ^]^

For the electronic microscopic measurement of GBM, imaging, and analysis of GBM were done in cooperation with the Institute of Pathology IPH at Heidelberg University Hospital. Briefly, kidneys were detached from adult zebrafish and fixed in 3% glutaraldehyde in cacodylate (0.1 m, pH 7.4), cut into 1 mm^3^ pieces, washed in buffer, and then post‐fixed in 1% aqueous osmium tetroxide for 1 h. Then, they were rinsed in water, dehydrated with ethanol solutions, transferred into propylene oxide, and embedded in epoxy resin (glycidether 100). Semithin (1 µm) and ultrathin sections (60–80 nm) were cut with an ultramicrotome (Reichert Ultracut E). Subsequently, semithin sections were stained with methylene blue and imaged by a light microscope (Olympus). Ultrathin sections were stained with uranyl acetate and lead citrate and imaged by a transmission electron microscope (JEM 1400), with a 2k TVIPS CCD camera (TemCam F216) at × 3000–× 10 000 magnification.

### Enzyme Activity Assay

At 5dpf, 120 larvae were anesthetized with 0.003% tricaine and immediately frozen in liquid nitrogen as a sample. The concentrations of RCS for the measurement of maximal activity (Vmax) for ALDH were determined from Michalis‐mention kinetics, as previously described.^[^ ^85^ ^]^ The concentration for each of the RCS used in this study were as follows: 5 mm AA 10 mm ABAL, 10 mm BAL, 2 mm MG,^[^ ^15^, ^86^ ^]^ 10 mm MDA, 4 mm 4HNE,^[^ ^15^ ^]^ 10 mm (2E)−2‐Hexadecenal and 10 mm tt‐DDE.

### Molecular Docking Analysis

To analyze the binding affinities and modes of interaction between tt‐DDE and insulin receptors, Autodock Vina v.1.1.2, a computational tool, was used for protein‐ligand docking.^[^ ^87^, ^88^ ^]^ The molecular structure of tt‐DDE and linsitinib was obtained from the PubChem Compound database (https://pubchem.ncbi.nlm.nih.gov/). 3D coordinates of the insulin receptor (with PDB ID, 1irk and resolution, 2.1 Å) were sourced from the Protein Data Bank (http://www.rcsb.org/pdb/home/home.do).

### tt‐DDE and Downstream Metabolite Determination via High‐Pressure Liquid‐Chromatography Mass Spectrometry (HPLC/MS)

Zebrafish larvae (40 pooled larvae/sample; ≈15 mg), liver and muscle samples were extracted for target quantification the following way: 20 × the volume of ice‐cold Methanol/H_2_O (80/20; v/v) containing 0.1% formic acid and 10 µm of 4‐hydroxy Nonenal‐d3 as internal standard was added to grounded samples. Samples were shortly vortexed, sonicated for 30 s on ice (2–5 ×) and incubated on ice for 10 min. After centrifugation at 14 000 rpm for 10 min at 4 °C, the supernatant was transferred to a new tube. The methanol part was evaporated with N_2_‐gas, the remaining water part was shock‐frozen in liquid nitrogen and freeze‐dried to complete dryness. Dried samples were reconstituted in 30 µl of H_2_O containing 0.1% formic acid, centrifuged as above, transferred to MS‐vials and ready to be analyzed.

For target identification and relative quantification, 15 µl per sample were injected into an Infinity II Bio liquid chromatography system coupled to a 6495 C triple quadrupole mass spectrometer (Agilent Technologies). Metabolites were separated on an Atlantis T3 column (2.1 × 150 mm, 3 µm; Waters) by using the solvent system and gradient as described previously.^[^ ^40^ ^]^ The gas temperature of the mass spectrometer was set to 290 °C and the gas flow to 20 L mi^−1^n. The nebulizer was set to 45 psi. The sheath gas flow was set to 11 L mi^−1^n, with a temperature of 400 °C. The capillary voltage was set to 5000 V with a nozzle voltage of 500 V (both positive mode). The MS analysis was performed in positive ionization mode. Samples were not only screened for tt‐DDE itself, but also for its downstream metabolites 2,4‐decadienoic acid, for the two conjugation products of tt‐DDE‐cysteine and tt‐DDE‐glutathione, and for the two conjugation alcohols of Cys‐conjugated 2,4‐decen‐1‐ol and GSH‐conjugated 2,4‐decen‐1‐ol. All metabolites were screened by their mass‐to‐charge ratio, retention time and fragmentation pattern (five fragments each, Figure S4A, Supporting Information). tt‐DDE was CE‐optimized by using an authentic standard. Cys‐conjugated 2,4‐decen‐1‐ol was identified as described previously.^[^ ^40^ ^]^ The fragmentation pattern of the remaining metabolites was developed according to mentioned paper and CE‐optimized by test injections with sample material.

### Pharmacological Treatment of Zebrafish Embryos/Larvae

Fertilized eggs were transferred into 6‐well plate, 30 eggs in a well with 5 mL egg water. At 24 hpf, embryos’ chorion was removed with tweezers and then immersed in different substrates and rescue medicines. For substrates treatment and rescue experiment, 0–20 µmol tt‐DDE (W313505, Sigma‐Aldrich), 0–200 µmol ABAL (A44150, Sigma‐Aldrich), 0–400 µmol BAL (B3650, Sigma‐Aldrich), and 10 mmol L‐Carnosine (C9625; Sigma‐Aldrich) were dissolved in egg water. Medium was refreshed daily.

### RNA‐seq Analysis

Total RNA was isolated from *aldh9a1b^+/+^, aldh9a1b^−/−^*, and *aldh9a1b^+/+^* with 8 µmol tt‐DDE treatment larvae at 5dpf. Library construction and sequencing were performed with BGISEQ‐500 (Beijing Genomic Institution, www.bgi.com, BGI). Gene expression analysis was conducted by the Core Lab for microarray analysis, Center for Medical Research (ZMF). Sequencing analyses were performed as described previously.^[^ ^15^ ^]^ The RNA‐Seq datasets produced in this study are available at GEO (Gene Expression Omnibus, NIH) under the accession number: (https://www.ncbi.nlm.nih.gov/geo/query/acc.cgi?acc = GSE223340)

### Western Blot Analysis

For western blot analysis, 20 larvae/one adult organ were taken and incubated for 10 min with 2 mm Natrium‐Vanadate (2 mm) in 1 × PBS on ice to inhibit phosphatases. Then fish were homogenized and lysed in NP40 lysis buffer (150 mmol L^−1^ NaCl, 50 mmol L^−1^ Tris‐HCl, pH 7.4, 1% NP40, 10 mmol L^−1^ EDTA, 10% glycerol, and protease inhibitors) on ice for 30 min on a shaker. The lysate was diluted with 5× Laemmli buffer and boiled at 95 °C for 5 min. SDS‐PAGE was used for electrophoresis and 0.2 µm supported nitrocellulose membrane (Amersham) was used for transblotting. The membranes were blocked with 5% BSA solution and incubated with primary antibodies 1:1000 (anti‐Actin, Santa Cruz Biotechnology, sc‐47778; AKT, CST, 9272S; P‐AKT, CST, 4060P), followed by secondary HRP‐conjugated antibodies 1:1000 (for β‐actin, rabbit anti mouse, DAKO, P0260; for P‐AKT and AKT, Goat anti‐Rabbit, Dako, P0448). Visualization by enhanced chemiluminescence (ECL) was were done by Western Lightning Plus ECL reagent (PerkinElmer) on a Vilber Fusion Solo S imaging system.

### Whole‐Body Glucose Determination in Zebrafish Larvae

20 larvae were collected as one sample at 5dpf and snap‐frozen in liquid nitrogen. Samples were homogenized in assay buffer with a 20 G syringe. A glucose assay kit (MAK263, Sigma‐Aldrich) was used to determine the glucose level according to the manufacturer's instructions. The fluorometric intensity was detected by a plate reader (Tecan Infinite M200).

### Blood Glucose Measurement

Adult Zebrafish were transferred to single boxes and fasted overnight. For fasting glucose measurement, fish were directly euthanized in ice water for 2 min. Blood was collected from caudal vessels and blood glucose was measured with a glucometer (Freestyle Abbott).^[^ ^89^ ^]^ For 2‐hour postprandial glucose measurement, fish were fed with 0.5 g flake food and changed water after 1 h. Then, the blood was extracted and measured as described above.

### Metabolomic Analysis

100 zebrafish larvae and one organ per fish were collected as a sample. Detection was performed in cooperation with the Metabolomics Core Technology Platform (MCTP) of the Center for Organismal Studies (COS) of Heidelberg University via gas chromatography‐mass spectrometry (GC/MS).^[^ ^90^ ^]^ Then, generated data was preprocessed with ChromaTof v5.50 software (LECO Corporation, Michigan) by converting signal peak area to signal intensity per sample amount. GC/MS data was adjusted to Ribitol, while fatty acids were normalized to C17:0. Quality control was evaluated by PLS‐DA and correlation map using R software. Differential metabolites were screened by fold change analysis with a threshold of 1.5. Advanced analysis related to function or pathway was done based on the KEGG database by MetaboAnalyst 5.0 according to the suggested protocol.^[^ ^91^ ^]^

### Statistical Analysis

All the data in this study were repeated with more than three independent biological replicates and presented with mean with standard deviation. Unpaired Student's *t* test was used for the comparison of two groups. One‐way and two‐way ANOVA was applied to multiple groups with one or two variables. The Chi square test was used for the analysis of the survival rate. Statistical analysis was performed by GraphPad Prism 8. Significance was defined as ^*^ *p* < 0.05, ^**^ *p* < 0.01, ^***^ *p* < 0.001, ^****^ *p* < 0.0001.

## Conflict of Interest

The authors declare no conflict of interest.

## Author Contributions

X.Q. designed this study, performed experiments, analyzed data, and wrote the manuscript. K.B. and D.P.W. maintained the zebrafish line and performed experiments. B.L. generated *aldh9a1b* knockout zebrafish. Y.M. analyzed RNA‐seq data. M.B. and G.P. performed metabolome studies analyzed data and gave technological advice. J.M. and T.F. implemented and performed biochemical experiments. S.K. and I.F. established the protocol, conducted the determination of tt‐DDE, and provided conceptual advice. C.S. performed metabolomics analysis. I.H. performed histological analysis and electron microscopy of adult zebrafish kidneys. J.S. and P.P.N. provided conceptual support. J.K. conceived and designed this study and wrote the manuscript. J.K. was the guarantor of this work, had full access to all data of the study, and took responsibility for the integrity and the accuracy of the data and the data analysis.

## Supporting information

Supporting Information

## Data Availability

The data that support the findings of this study are available in the supplementary material of this article.
