# Supplementary material for: Impaired Detoxification of Trans, Trans‐2,4‐Decadienal, an Oxidation Product from Omega‐6 Fatty Acids, Alters Insulin Signaling, Gluconeogenesis and Promotes Microvascular Disease
Source: Adv Sci (Weinh). 2023 Dec 7;11(4):2302325. doi: 10.1002/advs.202302325 (PMC10811472; doi:10.1002/advs.202302325)
Supplement: Supplementary file 1 — Supporting Information [file ADVS-11-2302325-s001.pdf]

ADVANCED SCIENCE Open Access

Supporting Information

for *Adv. Sci.*, DOI 10.1002/advs.202302325

Impaired Detoxification of Trans, Trans-2,4-Decadienal, an Oxidation Product from Omega-6 Fatty Acids, Alters Insulin Signaling, Gluconeogenesis and Promotes Microvascular Disease

*Xin Qian, Stephan Klatt, Katrin Bennewitz, David Philipp Wohlfart, Bowen Lou, Ye Meng, Michael Buettner, Gernot Poschet, Jakob Morgenstern, Thomas Fleming, Carsten Sticht, Ingrid Hausser, Ingrid Fleming, Julia Szendroedi, Peter Paul Nawroth and Jens Kroll**

Impaired detoxification of trans, trans-2,4-decadienal, an oxidation product from omega-6 fatty acids, alters insulin signaling, gluconeogenesis and promotes microvascular disease

Xin Qian[1], Stephan Klatt[2,3], Katrin Bennewitz[1], David Philipp Wohlfart[1], Bowen Lou[1,a], Ye Meng[4], Michael Buettner[5], Gernot Poschet[5], Jakob Morgenstern[6], Thomas Fleming[6], Carsten Sticht[7], Ingrid Hausser[8], Ingrid Fleming[2,3], Julia Szendroedi[6], Peter Paul Nawroth[6], Jens Kroll[1*]

[1]Department of Vascular Biology, European Center for Angioscience (ECAS), Medical Faculty Mannheim, Heidelberg University, Mannheim 68167, Germany

[2]Institute for Vascular Signaling, Centre for Molecular Medicine, Goethe-University, Frankfurt am Main 60590, Germany

[3]The German Centre for Cardiovascular Research (DZHK), Partner site RheinMain, Frankfurt 60590, Germany

[4]Bone Marrow Transplantation Center, The First Affiliated Hospital, Zhejiang University School of Medicine, Hangzhou 310003, China

[5]Metabolomics Core Technology Platform, Centre for Organismal Studies, Heidelberg University, Heidelberg 69120, Germany

[6]Department of Internal Medicine I and Clinical Chemistry, Heidelberg University Hospital, Heidelberg 69120, Germany

[7]NGS Core Facility, Medical Faculty Mannheim, Heidelberg University, Mannheim 68167, Germany

[8]Institute of Pathology IPH, EM Lab, Heidelberg University Hospital, Heidelberg 69120, Germany

***Corresponding Author:**

Prof. Dr. Jens Kroll

European Center for Angioscience (ECAS) Dept. of Vascular Biology

Medical Faculty Mannheim, Heidelberg University Ludolf-Krehl-Str. 13-17, 68167

Mannheim, Germany Phone: +49 (0) 621 383 71455

Fax: + 49 (0) 621 383 9961

Email: jens.kroll@medma.uni-heidelberg.de

[a]Current address: Cardiovascular Department, the First Affiliated Hospital of Xi’an Jiaotong University, 277 West Yanta Road, Xi'an, 710061, Ch

A

```
CLUSTAL O(1.2.4) multiple sequence alignment

sp|Q802W2|A9A1B_DANRE      MALMRCLLPPGFYRTLYHPWTRCASSGTLQIKDPLNFWCGARVDLKDVKTKSEPVFEPAT 60
sp|P49189|AL9A1_HUMAN      -------------------------MSTGTFVVSQPLNYRGGARVEPADAS-GTEKAFEPAT 36
sp|Q9JLJ2|AL9A1_MOUSE      -------------------------MSTGTFVVSQPLNYRGGARVEPVDAS-GTEKAFEPAT 36
                                                     *:**: :.:***:  ****:  *..  :* .*****

sp|Q802W2|A9A1B_DANRE      GRVLCRLQTCGSAEVDAAVRNASAAFKVWRKLSGMERARVMLEAARLIEKRREEIAEMEV 120
sp|P49189|AL9A1_HUMAN      GRVIATFTCSGEKEVNLAVQNAKAAFKIWSQKSGMERCRILLEAARIIREREDEIATMEC 96
sp|Q9JLJ2|AL9A1_MOUSE      GRVIATFACSGEKEVNLAVENAKAAFKLWSKKSGLERCQVLLEAARIIKERKDEIATVET 96
                           ***:.  :   .*. **: **.**.****:*  : **:**.:::*****:*.:*.:*** :*

sp|Q802W2|A9A1B_DANRE      INNGKSITEARLDVDSARLSIEYFAGQATTLSGQHVQLPGGSFAYTRREPFGVCVGIGAW 180
sp|P49189|AL9A1_HUMAN      INNGKSIFEARLDIDISWQCLEYYAGLAASMAGEHIQLPGGSFGYTRREPLGVCVGIGAW 156
sp|Q9JLJ2|AL9A1_MOUSE      INNGKSIFEARLDVDTCWQCLEYYAGLAASMAGEHIQLPGGSFGYTRREPLGVCVGIGAW 156
                           ******* *****:*  .   .:**:** *::::*:*:*******.******:*********

sp|Q802W2|A9A1B_DANRE      NYPFQIAAWKSAPAIACGNSMVFKPSPLTPVTAVLLAEIYRQAGAPEGLFNVVQGGQETG 240
sp|P49189|AL9A1_HUMAN      NYPFQIASWKSAPALACGNAMVFKPSPFTPVSALLLAEIYSEAGVPPGLFNVVQGGAATG 216
sp|Q9JLJ2|AL9A1_MOUSE      NYPFQIACWKSAPALACGNAMIFKPSPFTPVSALLLAEIYTKAGAPPGLFNVVQGGAATG 216
                           *******.******:****:*:******:***:*:******  :**.* *********  **

sp|Q802W2|A9A1B_DANRE      SLLCLHPSVEKVSFTGSVPTGKKIMEMASRGVKAVTLELGGKSPLIIFEDTDLENAVRGA 300
sp|P49189|AL9A1_HUMAN      QFLCQHPDVAKVSFTGSVPTGMKIMEMSAKGIKPVTLELGGKSPLIIFSDCDMNNAVKGA 276
sp|Q9JLJ2|AL9A1_MOUSE      QFLCHHREVAKISFTGSVPTGVKIMEMSAKGVKPITLELGGKSPLIIFSDCNMENAVKGA 276
                           .:** * .* *:********* *****:::*:* :*************.* :::***:**

sp|Q802W2|A9A1B_DANRE      LMANFLSQGQVCSNGTRVFVQSSIVPQFLKEVVRRTKAISIGDPLLDETRMGALVSKAHL 360
sp|P49189|AL9A1_HUMAN      LMANFLTQGQVCCNGTRVFVQKEILDKFTEEVVKQTQRIKIGDPLLEDTRMGPLINRPHL 336
sp|Q9JLJ2|AL9A1_MOUSE      LMANFLTQGQVCCNGTRVFVQKEIADKFINEVVKQTQKIKLGDPLLEDTRMGPLINAPHL 336
                           ******:*****.*********..*  :* :***::*: *.:*****::**** *:.  **

sp|Q802W2|A9A1B_DANRE      DKVLRYVEQAKNEGAQVLCGGEPFSPADPKLKDGYYMTPCVLDSCTDDMTCVKEEIFGPV 420
sp|P49189|AL9A1_HUMAN      ERVLGFVKVAKEQGAKVLCGGDIYVPEDPKLKDGYYMRPCVLTNCRDDMTCVKEEIFGPV 396
sp|Q9JLJ2|AL9A1_MOUSE      ERVLGFVKLAKEQGATVLCGGEVYVPEDPKLKHGYYMTPCILTNCRDDMTCVKEEIFGPV 396
                           ::**  :*: **::** *****: : * *****.**** **:* .* **************

sp|Q802W2|A9A1B_DANRE      MSVLTFDTEDEVLRRANDSDLGLAAGVFTKDVKRAHRVIENLQAGSCFINNYNITPVEVP 480
sp|P49189|AL9A1_HUMAN      MSILSFDTEAEVLERANDTTFGLAAGVFTRDIQRAHRVVAELQAGTCFINNYNVSPVELP 456
sp|Q9JLJ2|AL9A1_MOUSE      MSILTFGTEAEVLERANDTTFGLAAGVFTRDIQRAHRVAAELQAGTCYINNYNVSPVELP 456
                           **:*:*.** ***.****: :********:*::******   :****:*:******::***:*

sp|Q802W2|A9A1B_DANRE      FGGFKASGIGRENGQVTIEFYSQLKTVVVEMGDVDSLF          518
sp|P49189|AL9A1_HUMAN      FGGYKKSGFGRENGRVTIEYYSQLKTVCVEMGDVESAF          494
sp|Q9JLJ2|AL9A1_MOUSE      FGGYKKSGFGRENGRVTIEYYSQLKTVCVEMGDVESAF          494
                           ***:* **:*****:****:******* ******:* *
```

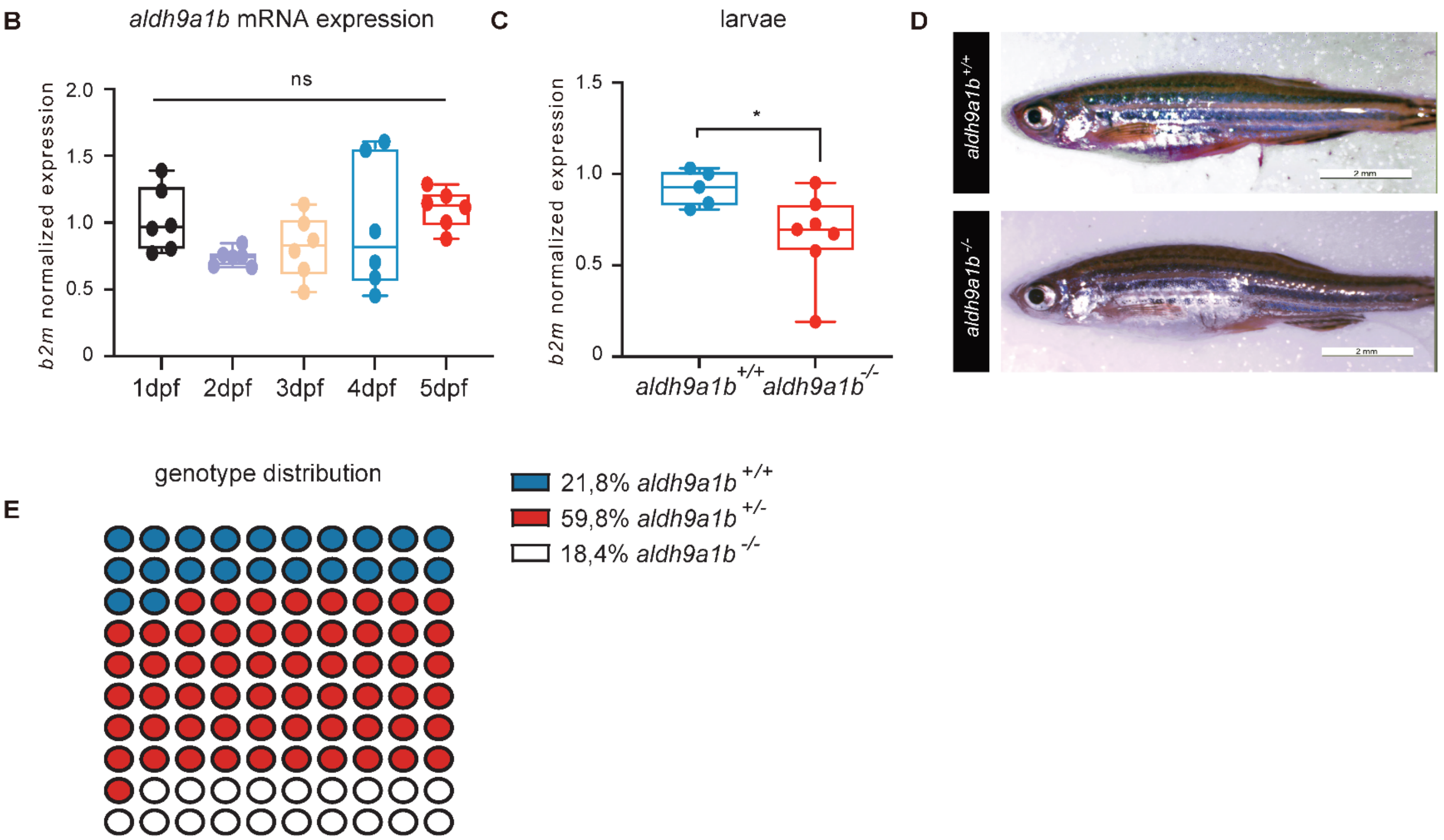

Figure.S1 Generation and validation of *aldh9a1b$^{-/-}$* zebrafish using CRISPR-Cas9 technology (A) Amino acid alignment of *aldh9a1* showed high similarity, same active site (red frame) and NAD binding site (blue frame) between zebrafish, human and mouse. (B) *aldh9a1b* mRNA expression were not changed by indicated developmental stages, n=6. (C) *aldh9a1b* mRNA expression was significantly decreased in *aldh9a1b$^{-/-}$* larvae, n=5 and n=7. (D) Representative images of *aldh9a1b$^{+/+}$* and *aldh9a1b$^{-/-}$* 12 months old zebrafish showed normal morphology. (E) Zebrafish number among different genotypes was in line with the Mendelian Inheritance in the first generation of F2: *aldh9a1b$^{+/+}$*=52, *aldh9a1b$^{+/-}$*=143, *aldh9a1b$^{-/-}$*=44. mRNA Expression was quantified by RT-qPCR and normalized to b2m. Each datapoint in this figure represented 20 larvae. The bars indicate mean±SD values. Statistical analysis was performed by Student's t-test, one-way ANOVA and chi-square test, ns, not significant; $*p < 0.05$.

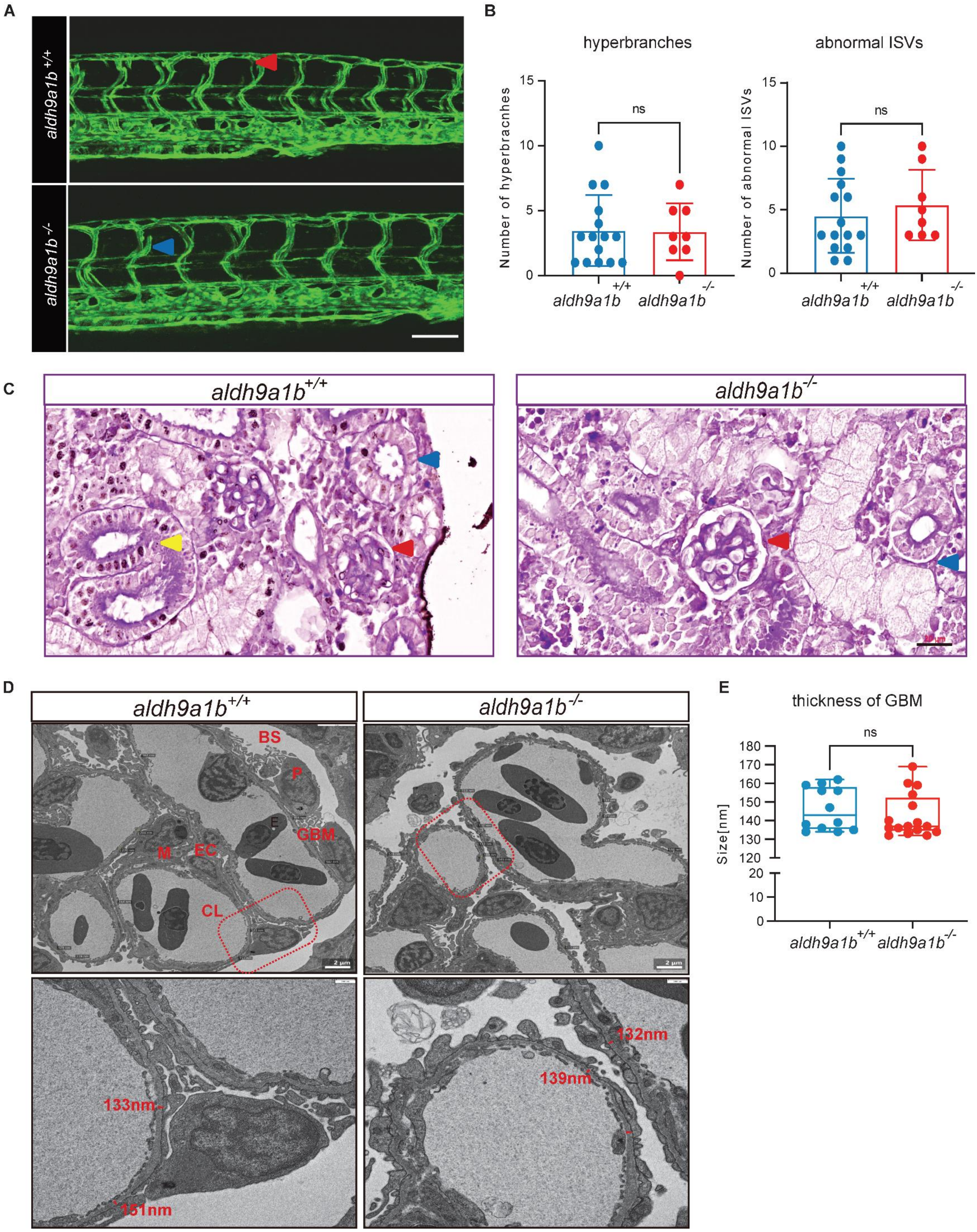

Figure.S2 Unaltered trunk vasculature and kidney morphology in *aldh9a1b*$^{-/-}$ zebrafish (A) Representative confocal images of trunk vasculature in *aldh9a1b*$^{+/+}$ and *aldh9a1b*$^{-/-}$ larvae at 4dpf. Red arrow, hyperbranches; bule arrow, abnormal

ISVs. White scale bar=100μm. (B) Quantification of hyperbranches and abnormal ISVs showed unchanged trunk vasculature between *aldh9a1b*$^{+/+}$ and *aldh9a1b*$^{-/-}$ larvae at 4dpf. Each datapoint represented one larva, n=15 and n=8. (C) Representative Periodic acid–Schiff (PAS) staining showed normal gross structure of *aldh9a1b*$^{+/+}$ and *aldh9a1b*$^{-/-}$ kidneys. Yellow arrow, PAS-positive hyaline droplets; blue arrow, renal tubule; red arrow, glomeruli. Black scale bar, 20μm. (D) Representative electron micrographs of glomeruli of *aldh9a1b*$^{-/-}$ and *aldh9a1b*$^{+/+}$ zebrafish. Scale bar, 2μm and 500nm. (E) *aldh9a1b*$^{-/-}$ glomeruli displayed normal thickness of GBM compared to *aldh9a1b*$^{+/+}$. The bars indicate mean±SD values. Statistical analysis was performed by Student's t-test. ns, not significant. GBM, glomerular basement membrane; BS, bowman space; P, podocyte; M, mesangium; EC, endothelial cell; CL, capillary lumen; E, erythrocyte.

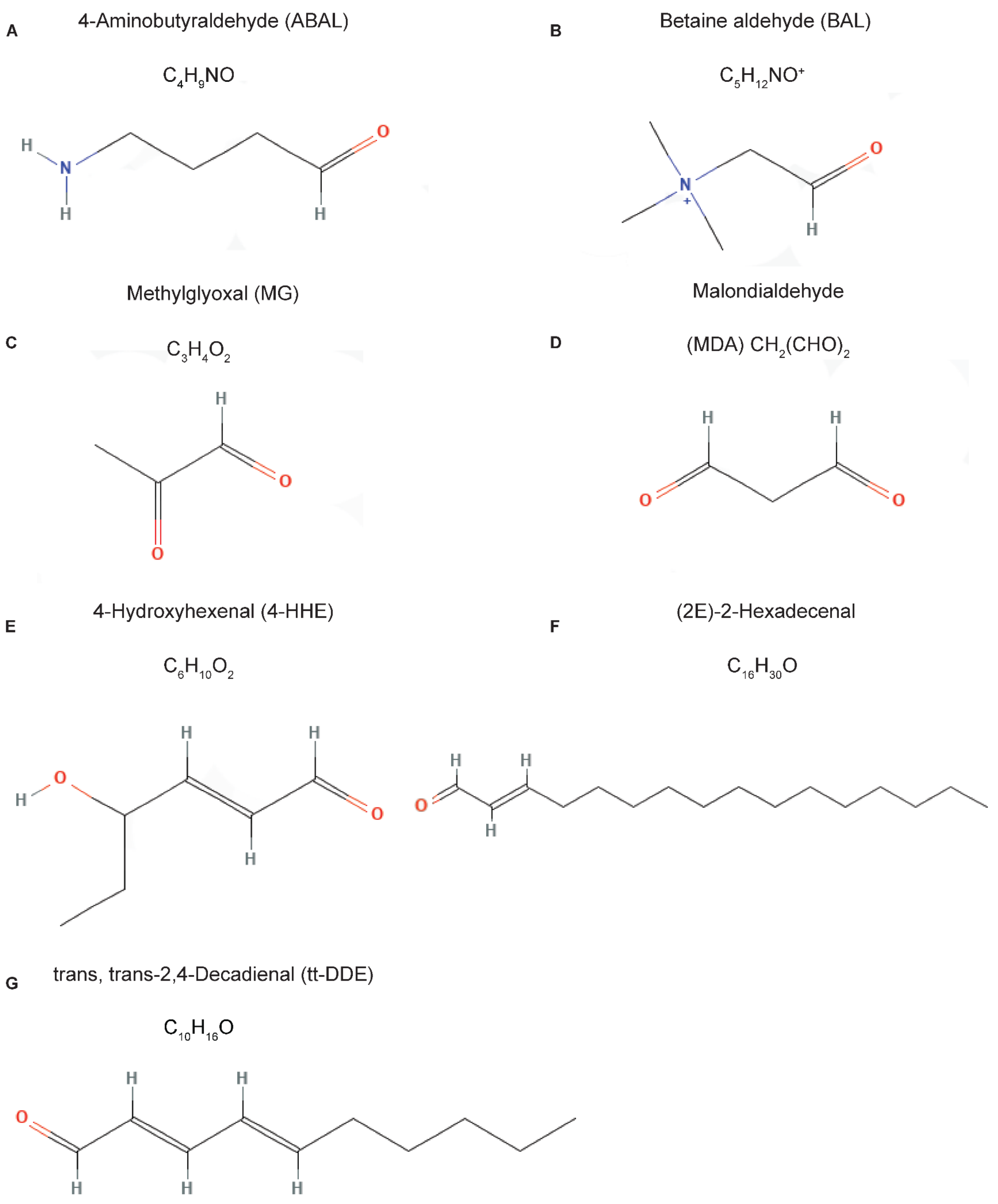

Figure.S3 Illustration of structure of analyzed substrates for Aldh9a1b (A) 4-Aminobutyraldehyde (ABAL) (B) Betaine aldehyde (BAL) (C) Methylglyoxal (MG) (D) Malonaldehyde (MDA) (E) 4-Hydroxyhexenal (4-HHE) (F) (2E)-2-Hexadecenal (G) trans, trans-2,4-Decadienal (tt-DDE)

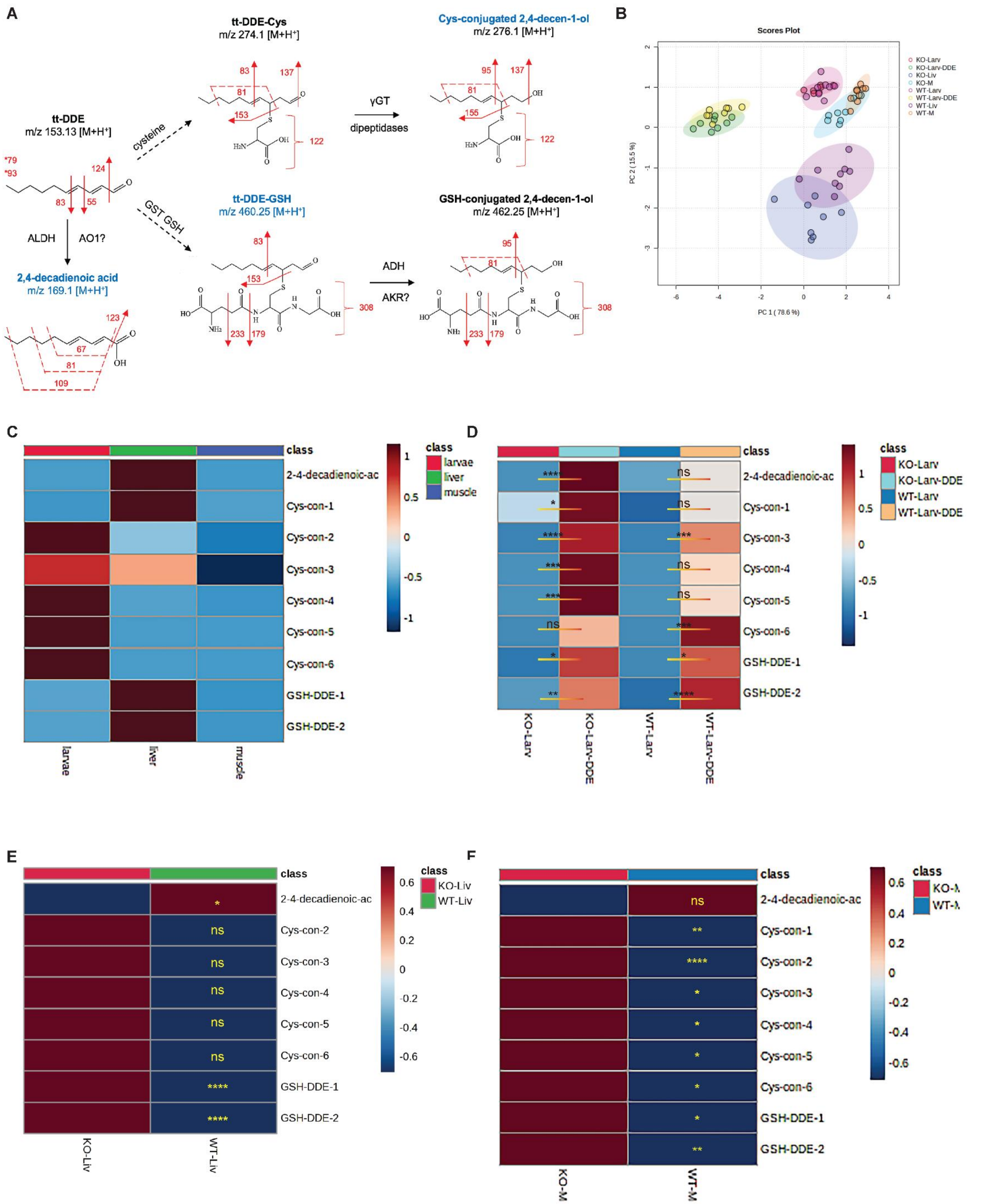

Figure S4. tt-DDE metabolites were significantly changed induced by *aldh9a1b* knockout and tt-DDE treatment. (A) The fragmentation pattern/selected fragments which were identified via HPLC/MS are shown (*unknown fragment location). The fragmentation pattern of tt-DDE was CE-optimized on the 6495C QQQ with the use of an authentic standard. Three metabolites that can be detected were highlighted in blue. (B) PCA analysis was conducted to visualize the

differentiation among the analyzed groups. (C) A heatmap illustrates the abundance level of tt-DDE metabolites in different organs. Larvae exhibited higher levels of cyscon2-cyscon6, the liver was notably enriched in cys-con-1 and GSH-DDE, while muscle predominantly contained cys-con-2 and cys-con-3. Each group contains all the respective samples with n=17 in liver and muscles, and n=31 in the larvae group. (D-F) tt-DDE metabolites were significantly changed induced by *aldh9a1b* knockout and tt-DDE treatment. n=9 and 8 in liver and muscle, n=8 and n=7 in larvae. Statistical analysis was performed by Student's t-test and one-way ANOVA. ns=not significant, *$p < 0.05$, **$p < 0.01$, ***$p < 0.001$, ****$p < 0.0001$. MS, Mass spectrometry; tt-DDE, trans, trans-2,4-decadienal; CYS-con2, Cys-conjugated 2,4-decen-1-ol; WT, *aldh9a1b*$^{+/+}$; KO, *aldh9a1b*$^{-/-}$; Liv, liver; M, muscles.

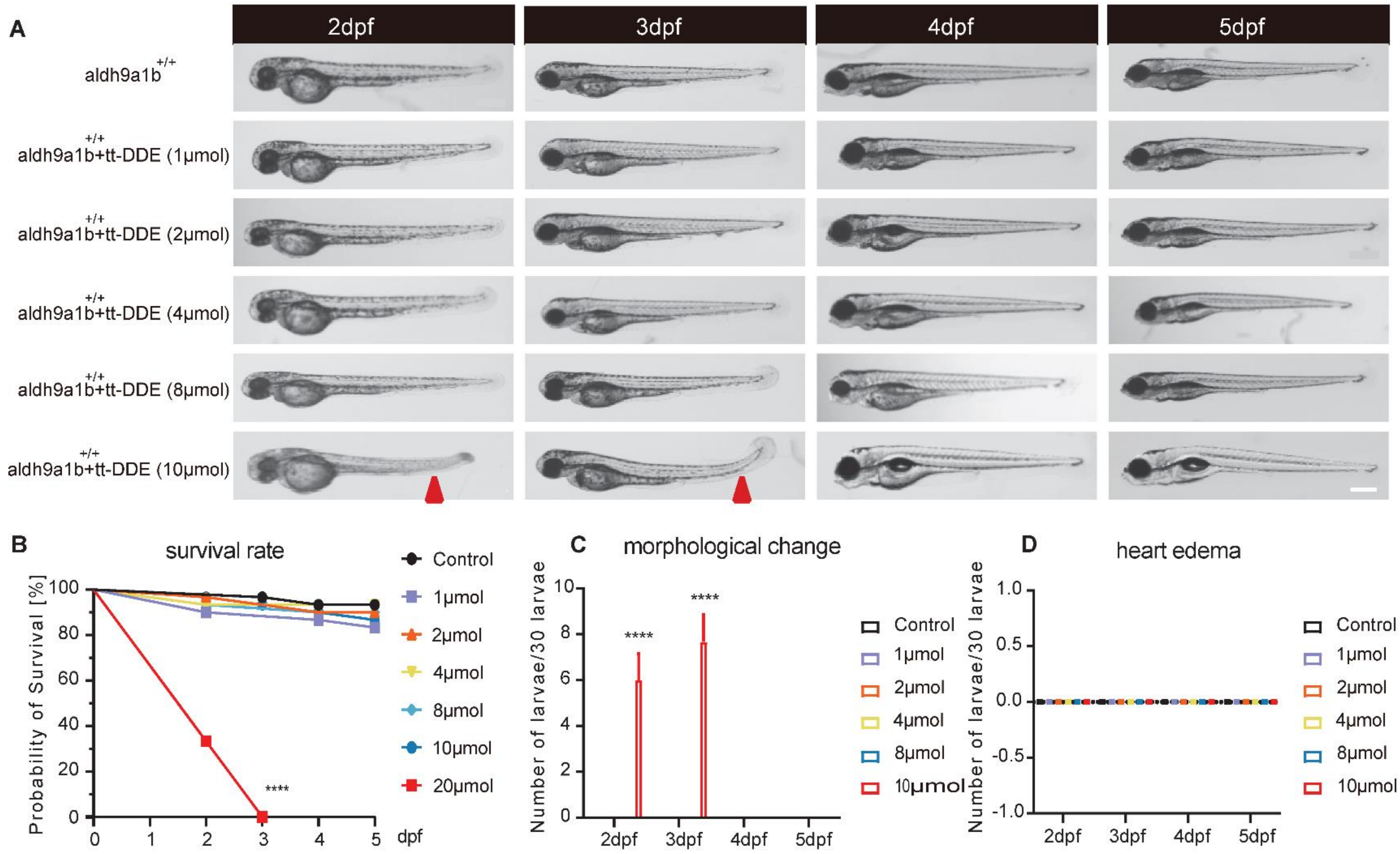

Figure.S5 Morphological alterations in *aldh9a1b*$^{+/+}$ larvae treated with tt-DDE (A) Representative microscopic images of zebrafish larvae between 2dpf and 5dpf with 0–10μmol tt-DDE treatment. White bar, 200μm. Red arrow, morphological change. (B) Quantification of survival rates showed lethality of 20μmol tt-DDE treated zebrafish larvae. (C) Quantification of morphological change showed significant alterations in the 2dpf and 3dpf larvae treated with 10μmol tt-DDE. (D) Quantification of heart edema showed normal heart development between different groups. The bars indicate mean±SEM values. Statistical analysis was performed by one-way ANOVA, two-way ANOVA and logrank test. ns = not significant, ****$p < 0.0001$. tt-DDE, trans, trans-2,4-decadienal.

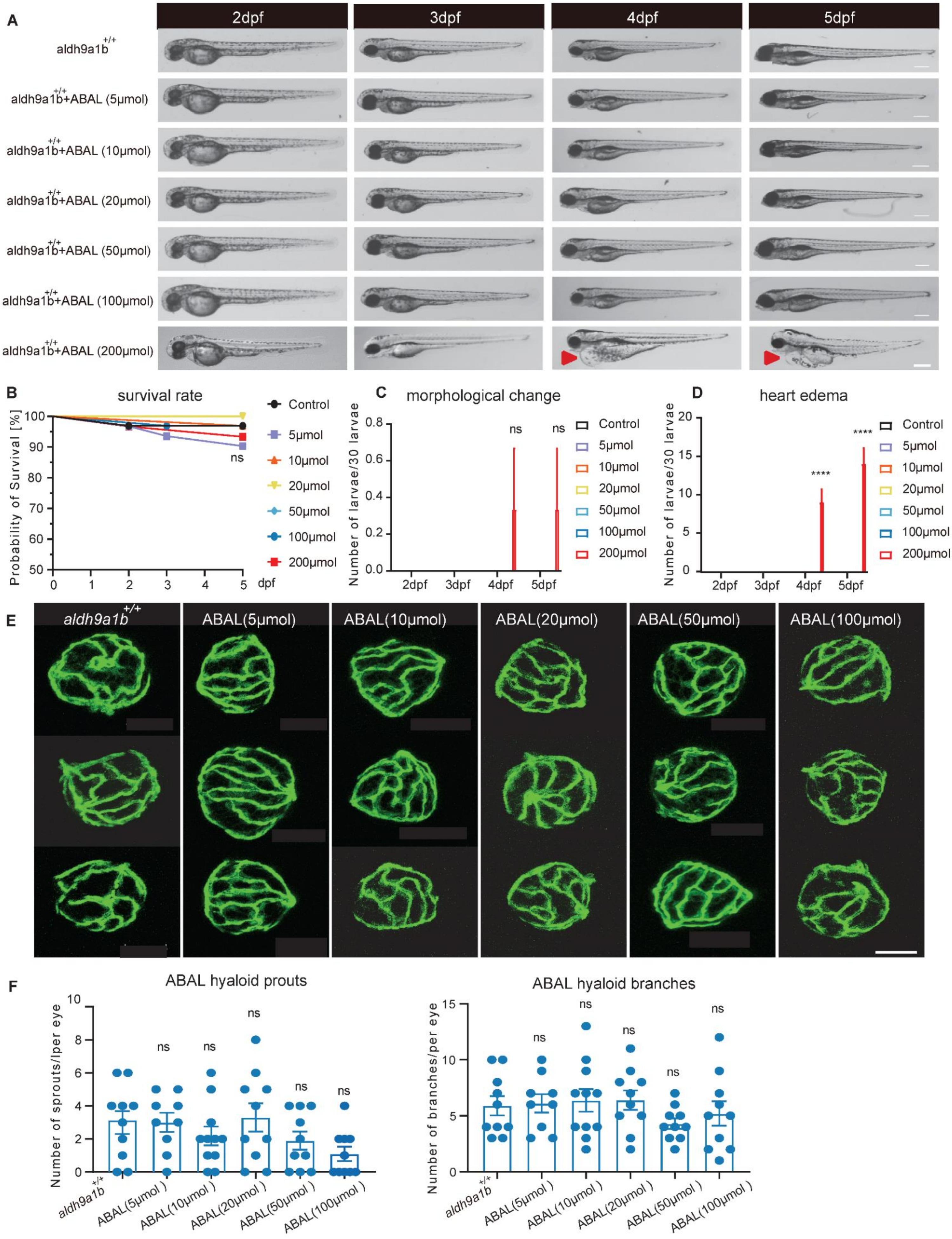

Figure.S6 Morphological and hyaloid vascular alterations in *aldh9a1b*$^{+/+}$ larvae treated with ABAL (A)

Representative microscopic images of *aldh9a1b*$^{+/+}$ zebrafish larvae between 2dpf and 5dpf with 0 – 200µmol ABAL treatment. Red arrow, heart edema. White bar, 200µm. (B) Quantification of survival rates showed no significant change between different groups. (C) Quantification shows normal morphology across all the groups. (D) Quantification of heart edema showed significant increase in *aldh9a1b*$^{+/+}$ larvae at 4dpf and 5dpf with 200µmol ABAL treatment. (E) Representative confocal images of hyaloid vasculature showed vascular alterations in *aldh9a1b*$^{+/+}$ zebrafish larvae treated with 0–200µmol ABAL at 5dpf. White scale bar = 50µm. (F) Quantification of hyaloid branchpoints and sprouts formation showed no significant alterations across all the ABAL concentrations. One datapoint means one hyaloid per larva. The bars indicate mean±SEM values. Statistical analysis was performed by one-way ANOVA, two-way ANOVA and logrank test. ns = not significant, ****$p < 0.0001$. ABAL, 4-aminobutyraldehyde.

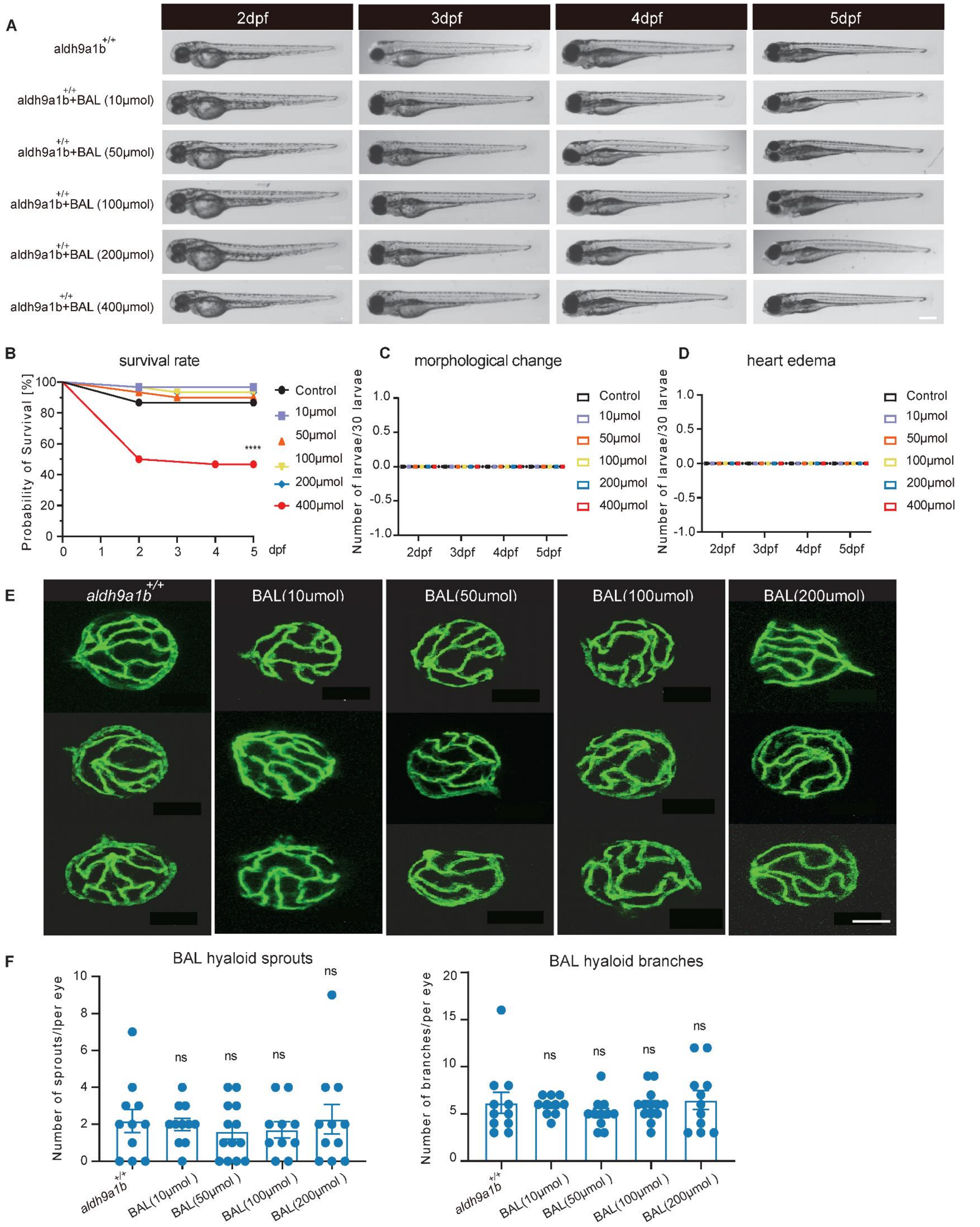

Figure.S7 Morphological and hyaloid vascular analysis in *aldh9a1b*$^{+/+}$ larvae treated with BAL (A) Representative unaltered microscopic images of zebrafish larvae between 2dpf and 5dpf with 0–400μmol BAL treatment. White bar,

200μm. (B) Quantification of survival rates showed higher lethality of zebrafish larvae treated with 400μmol BAL. (C) Quantification of morphology change showed no significant change between different groups. (D) Quantification of heat edema showed normal heart development compared to *aldh9a1b*$^{+/+}$ group. (E) Representative confocal images of hyaloid vasculature showed vascular alterations in zebrafish larvae treated with 0–200μmol BAL at 5dpf. White scale bar = 50μm. (F) Quantification of no significant change of hyaloid branchpoints and sprouts across different BAL concentrations. One datapoint means one hyaloid per larva. The bars indicate mean±SEM values. Statistical analysis was performed by one-way ANOVA, two-way ANOVA and logrank test. ns = not significant, ****$p < 0.0001$. BAL, betaine aldehyde.

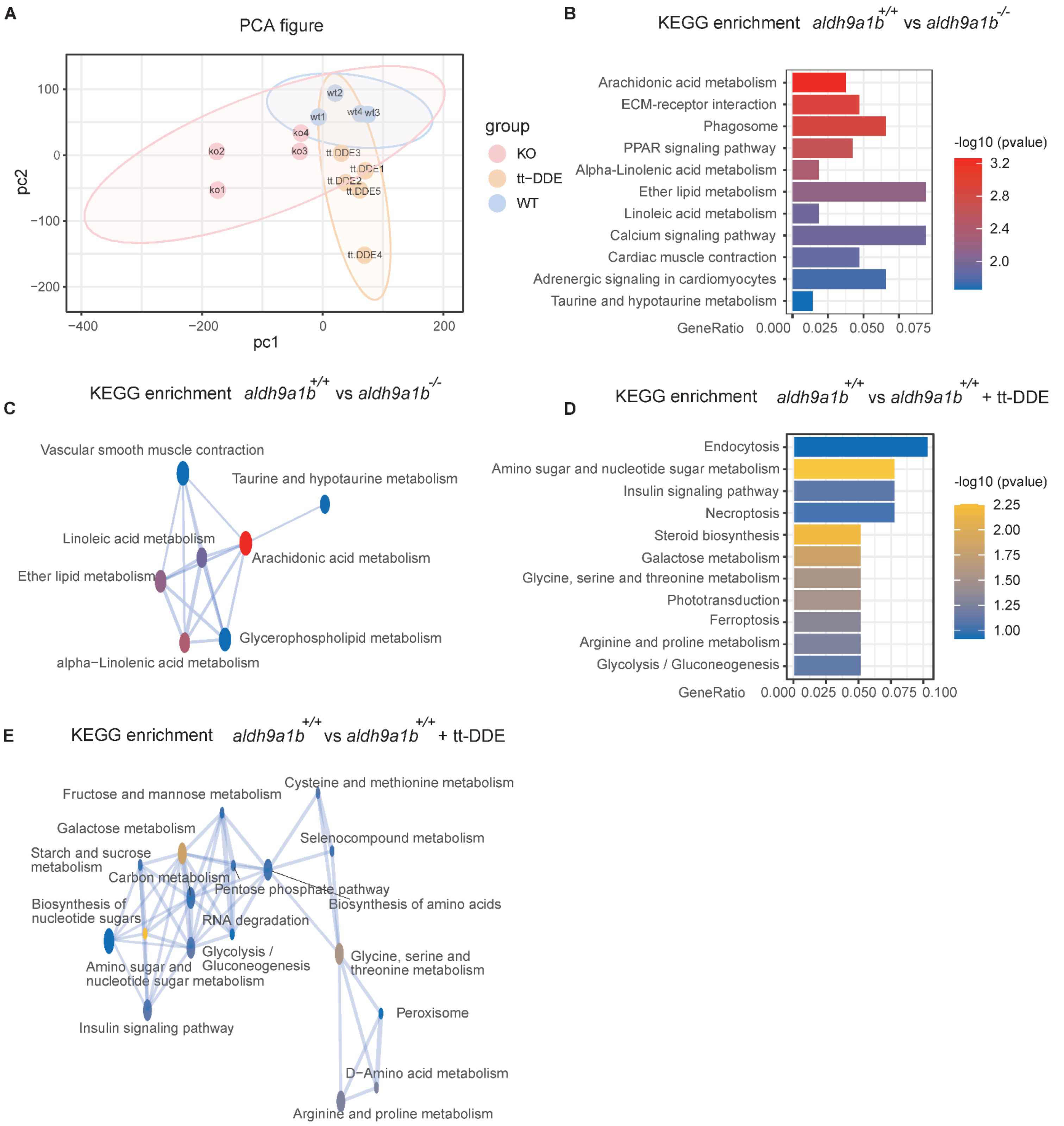

Figure.S8 RNA-seq analysis of *aldh9a1b* larvae at 5dpf (A) PCA figure showed different groups of RNA-seq. (B) KEGG enrichment analysis of differential genes showed top altered pathways in *aldh9a1b*$^{-/-}$ compared to *aldh9a1b*$^{+/+}$. (C) Emapplot of KEGG enrichment analysis showed the interaction of altered pathways in *aldh9a1b*$^{-/-}$ mutants. (D) KEGG enrichment analysis of differential genes showed top altered pathways in tt-DDE treated *aldh9a1b*$^{+/+}$ larvae compared to *aldh9a1b*$^{+/+}$ larvae. (E) Emapplot of KEGG enrichment analysis showed the interaction of altered pathways in the tt-DDE treated group.

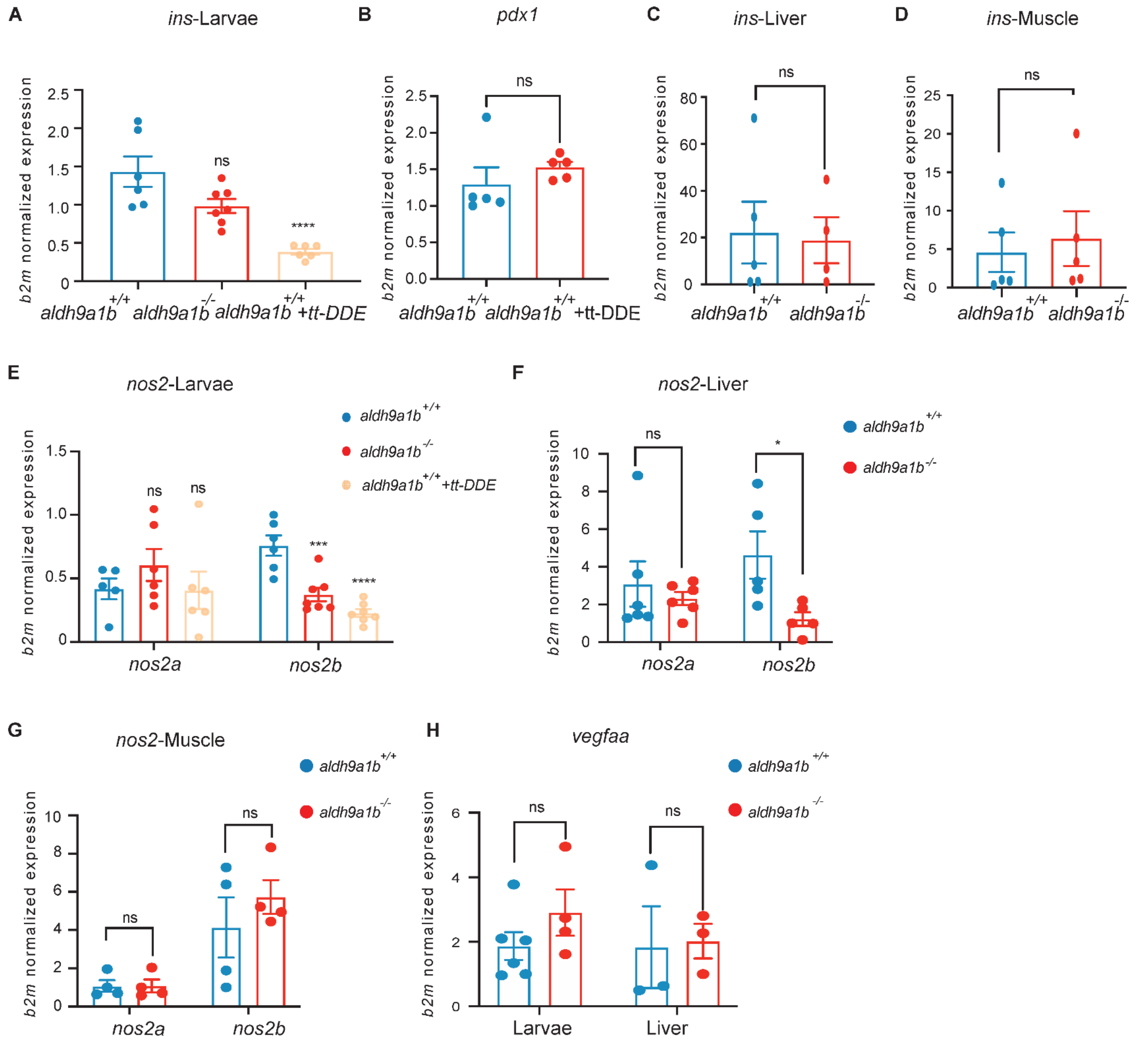

Figure.S9 Altered insulin receptor signaling in *aldh9a1b*$^{-/-}$ and in tt-DDE treated *aldh9a1b*$^{+/+}$ zebrafish larvae (A) mRNA expression of *ins* was significantly decreased in tt-DDE treated *aldh9a1b*$^{+/+}$, and slightly reduced in *aldh9a1b*$^{-/-}$ larvae, n=6 and 7. (B) mRNA expression of *pdx1* was not changed in tt-DDE treated *aldh9a1b*$^{+/+}$ compared to *aldh9a1b*$^{+/+}$ larvae, n=5. (C-D) mRNA expression of *ins* were not altered in the livers (C) and muscles (D) of *aldh9a1b*$^{-/-}$ zebrafish, n=5. (E) *nos2a* mRNA level was not changed across three groups, while *nos2b* was significantly decreased in *aldh9a1b*$^{-/-}$ and tt-DDE treated larvae, n=6 and 7. (F-G) *nos2a* and *nos2b* were not altered in livers and muscles of *aldh9a1b*$^{-/-}$ zebrafish,

n=6 and 4. (H) mRNA expression of *vegfaa* was not changed in the larvae and livers of *aldh9a1b*$^{-/-}$ zebrafish, n=6 and 4. mRNA Expression was quantified by RT-qPCR and normalized to b2m. Each datapoint in this figure represented 20 larvae or one fish. The bars indicate mean±SEM values. Statistical analysis was performed by Student's t-test, one-way ANOVA. ns, not significant; ****$p < 0.0001$. INS, insulin; PDX1, pancreatic and duodenal homeobox 1; NOS2, nitric oxide synthase 2; VEGFAa, vascular endothelial growth factor Aa.

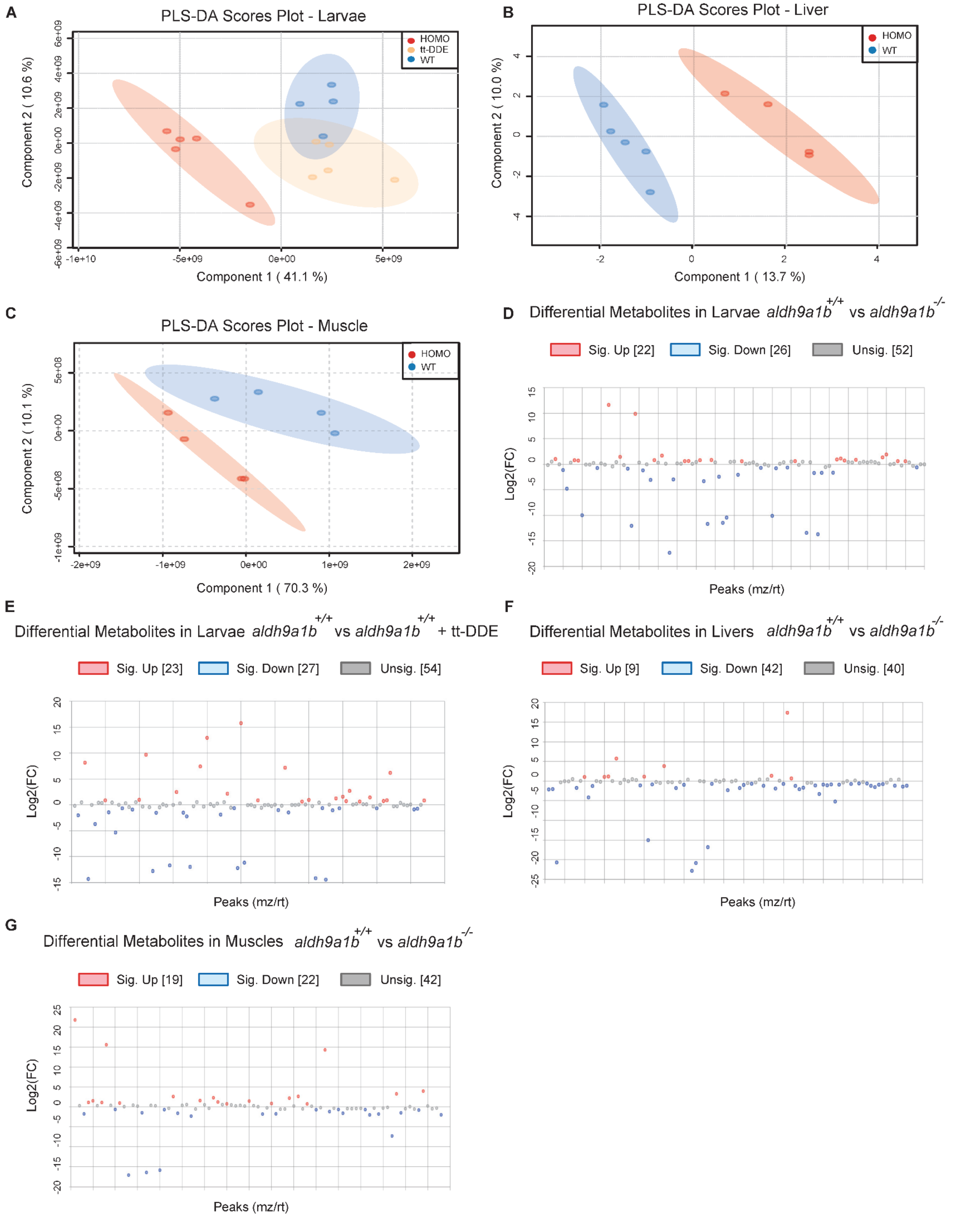

Figure.S10 Altered glucose metabolism in *aldh9a1b*$^{-/-}$ and tt-DDE treated zebrafish (A-C) PLS-DA scores plot showed different groups in the larvae (A), livers (B) and muscles (C) of adult zebrafish. (D-G) Fold change analysis displayed differential metabolites induced by *aldh9a1b*$^{-/-}$ or tt-DDE by comparison to *aldh9ab*$^{+/+}$.

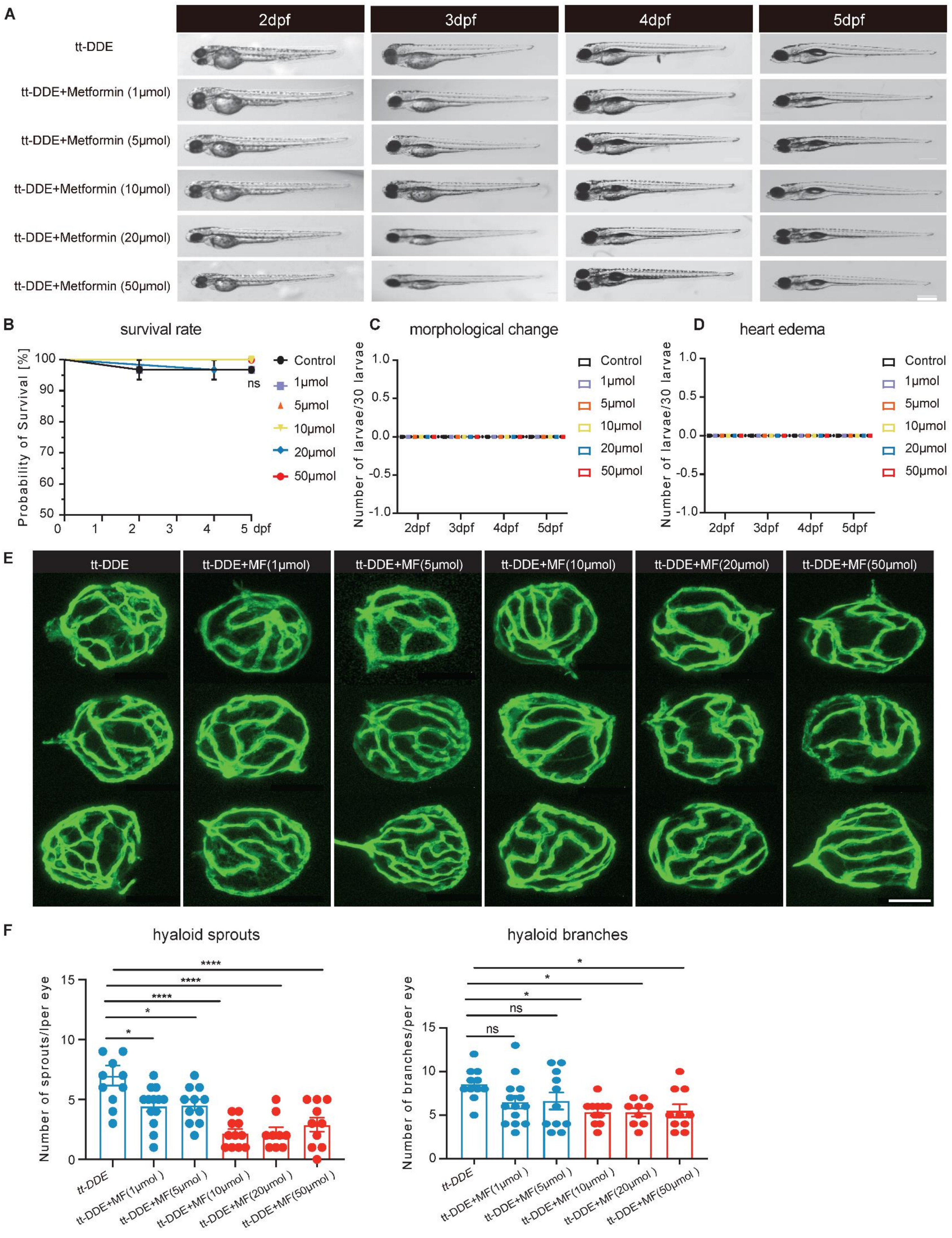
A
2dpf
3dpf
4dpf
5dpf
tt-DDE
tt-DDE+Metformin (1μmol)
tt-DDE+Metformin (5μmol)
tt-DDE+Metformin (10μmol)
tt-DDE+Metformin (20μmol)
tt-DDE+Metformin (50μmol)
B
survival rate
Probability of Survival [%]
ns
dpf
Control
1μmol
5μmol
10μmol
20μmol
50μmol
C
morphological change
Number of larvae/30 larvae
D
heart edema
E
tt-DDE
tt-DDE+MF(1μmol)
tt-DDE+MF(5μmol)
tt-DDE+MF(10μmol)
tt-DDE+MF(20μmol)
tt-DDE+MF(50μmol)
F
hyaloid sprouts
Number of sprouts/per eye
hyaloid branches
Number of branches/per eye
tt-DDE
tt-DDE+MF(1μmol)
tt-DDE+MF(5μmol)
tt-DDE+MF(10μmol)
tt-DDE+MF(20μmol)
tt-DDE+MF(50μmol)

Figure.S11 Morphological and hyaloid vascular rescue in *aldh9a1b*$^{+/+}$ larvae co-treated with tt-DDE and metformin (A) Representative microscopic images of zebrafish larvae between 2dpf and 5dpf co-treated with 8μmol tt-DDE and 0-50μmol MF. White bar, 200μm. (B) Quantification of survival rates showed no significant change between these groups. (C) Quantification of morphology change showed normal morphology between different groups. (D) Quantification of heat edema showed normal heart development across all the groups. (E) Representative confocal images of hyaloid vasculature showed beneficial effects in zebrafish larvae co-treated with 8 μmol tt-DDE and 0-50μmol MF at 5dpf. White scale bar=50μm. (F) Quantification of hyaloid branchpoints and sprouts formation showed increased angiogenic vasculature caused by tt-DDE treatment, which can be rescued by 1-50μmol MF. One datapoint means one hyaloid per larva. The bars indicate mean ± SEM values. Statistical analysis was performed by one-way ANOVA, two-way ANOVA and logrank test. ns = not significant, *$p < 0.05$. MF, metformin.

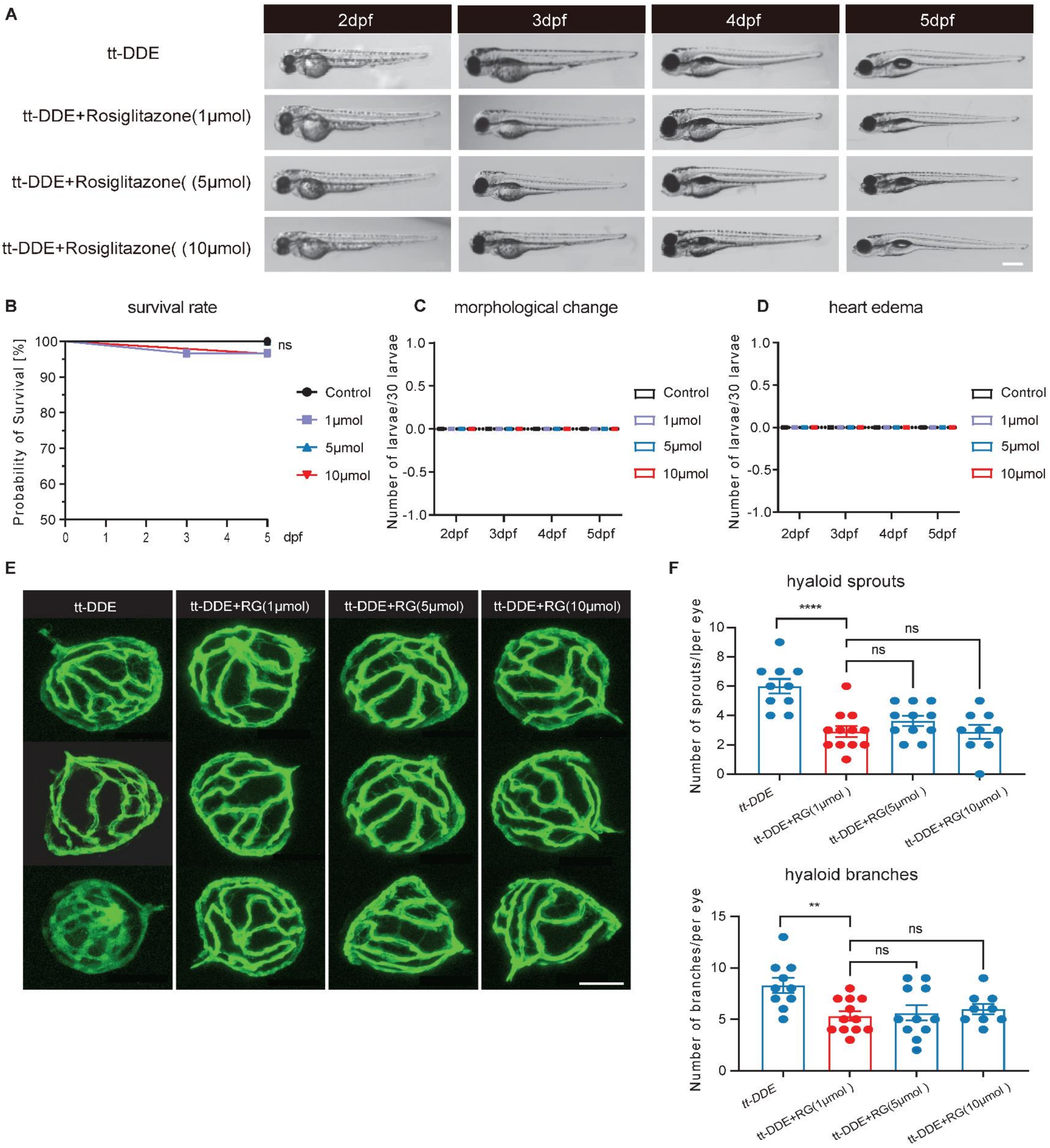

Figure.S12 Morphological and hyaloid vascular rescue in *aldh9a1b*$^{+/+}$ larvae co-treated with tt-DDE and rosiglitazone (A) Representative microscopic images of zebrafish larvae between 2dpf and 5dpf co-treated with 8μmol tt-DDE and 0-10μmol RG. White bar, 200μm. (B) Quantification of survival rates showed no significant change between these groups. (C) Quantification of morphology change showed no significant alterations across all the groups. (D) Quantification of heat edema showed normal heart morphology between different groups. (E) Representative confocal images of hyaloid vasculature showed rescue of vascular alterations in zebrafish larvae co-treated with 8μmol tt-DDE and 0-10μmol RG at 5dpf. White scale bar=50μm. (F) Quantification of hyaloid branchpoints and sprouts formation showed increased angiogenic vasculature caused by tt-DDE, which can be rescued by 1-10μmol rosiglitazone. One datapoint

means one hyaloid per larva. The bars indicate mean±SEM values. Statistical analysis was performed by one-way ANOVA, two-way ANOVA and logrank test. ns = not significant, **p < 0.01, ****p < 0.0001. RG, rosiglitazo

Primer Name	Primer Sequence	Primer Name	Primer Sequence
9a1b_genotyping_forward	CCTGCCATCGCTTGTGGTAT	g6pc1a.1_qpcr_forward	GCTGCACCATACGAGATGGA
9a1b_genotyping_reverse	TCTTTCCTGTGGGCACACTTC	g6pc1a.1_qpcr_reverse	TCACCAAACAGCACCCACTT
9a1b_qpcr_forward	CTATGGTGTTCAAGCCGTCTC	fbp1a_qpcr_forward	CATCTGTATGGGATTGCTGG
9a1b_qpcr_reverse	CACCGAAGGGTGAAGACAGAG	fbp1a_qpcr_reverse	TTACCCCGTCTATCTGGCTC
b2m_qpcr_forward	GCAACGCTCTTTGTGAGGTG	pck1_qpcr_forward	GTGAACTGAACCGAGACCTG
b2m_qpcr_reverse	AACCACTGAACACGGACCTC	pck1_qpcr_reverse	AGCACTTGAGAGCAAACGAT
ins_qpcr_forward	GCCCAACAGGCTTCTTCTACAAC	ugp2a_qpcr_forward	CTGACGGGAGAGAATGAGGA
ins_qpcr_reverse	GCAGATTTAGGAGGAAGGAAACCC	ugp2a_qpcr_reverse	GTCTTGGGGTTGACAATGAT
insra_qpcr_forward	AGAGGCCAGCGAGCTCTAC	gys2 _qpcr_forward	AAATCTTCCCCTGGCGACTA
insra_qpcr_reverse	CACTTGTGTGGGGGCTCT	gys2 _qpcr_reverse	AAAGTTGTTGGTTTTGGCGG
insrb_qpcr_forward	GCCTCTGCGGATCACTACAT	glut2_qpcr_forward	GCAGAAGAACCCTCACTC
insrb_qpcr_reverse	CTCCTGCGTGGTCTTGAAC	glut2_qpcr_reverse	TCTCCGCCACAATAAACC
irs1_qpcr_forward	ACTACTCTTTGCCCCGCTCA	pdx1_qpcr_forward	ACACGCACGCATGGAAAGGACA
irs1_qpcr_reverse	TCGTCCGTTGGTTACTGTCG	pdx1_qpcr_reverse	GCGGGCGCGAGATGTATTTGTT
hk1_qpcr_forward	ATGATAGCGGCACAGCTTCT	NOS2a_qpcr_forward	TGCCGTCATTCACAGCTATC
hk1_qpcr_reverse	GTTGGTGTCTCGTGCCAATC	NOS2a_qpcr_reverse	ACCAGCCAGACCCAATCC
gck_qpcr_forward	AATCACCGCTGACCTGCTAT	NOS2b_qpcr_forward	AACGGCATCATGAACTGTTG
gck_qpcr_reverse	GCCACTTCACATACGCAATG	NOS2b_qpcr_reverse	TACATTGTAGTCCTCCATGCAAA
pklr_qpcr_forward	CAAAGGACACTTCCCTGTAGAG		
pklr_qpcr_reverse	GGACAACGAGGACGATAACG		

Table.S1 Primers List
